# Integrative multi-omics reveals that downregulation of HLA-DPA1/DPB1 drives macrophage immune-metabolic dysregulation in pediatric asthma

**DOI:** 10.3389/fimmu.2026.1835475

**Published:** 2026-06-03

**Authors:** Yuchen Wen, Zefan Du, Zhiyuan Zhong, Qiurong Yuan, Liangkang Lin, Ran Yao, Jiaying He, Qionghui Huang, Liang Li, Cheng Ouyang, Junbing Huang, Su Liu, Chun Chen

**Affiliations:** 1 Pediatric Laboratory, Department of Pediatrics, The Seventh Affiliated Hospital of Sun Yat-Sen University, Shenzhen, Guangdong, China; 2 Scientific Research Center, The Seventh Affiliated Hospital of Sun Yat-sen University, Shenzhen, Guangdong, China; 3 Department of Clinical Medical Laboratory, The Seventh Affiliated Hospital of Sun Yat-sen University, Shenzhen, Guangdong, China

**Keywords:** HLA-DPA1/HLA-DPB1, macrophage subsets, MHC class II antigen presentation, pediatric asthma, single-cell RNA sequencing

## Abstract

**Background:**

Pediatric asthma (PA) is a prevalent chronic respiratory disease. Emerging evidence suggests that dysregulated macrophage heterogeneity and immune-metabolic crosstalk contribute to disease pathogenesis, yet specific molecular nodes linking innate immune dysfunction to PA remain unidentified. This study aimed to identify and characterize immune checkpoint-related candidate key genes in PA.

**Methods:**

Bulk RNA-sequencing data from airway epithelium of PA patients (training set GSE152004) were analyzed for differential expression, followed by intersection with immune checkpoint-related genes. Four machine learning algorithms (SVM−RFE, Boruta, LASSO, and XGBoost) were applied to screen candidate key genes, which were further validated in an independent dataset (GSE65204). A nomogram was constructed to evaluate diagnostic value. Functional enrichment, immune infiltration, and regulatory network analyses were performed. *In vitro* IL-13 stimulation of bronchial epithelial cells and patient peripheral blood mononuclear cell samples were used for experimental validation. Single-cell RNA-seq data (GSE254127) were analyzed for cell typing, macrophage subclustering, pseudotime trajectory, and cell−cell communication.

**Results:**

HLA-DPA1 and HLA-DPB1 were identified as candidate key genes by consensus of all four algorithms. Both were significantly downregulated in PA and showed high diagnostic value (nomogram). Downregulation of HLA-DPA1/DPB1 correlated with attenuated antigen presentation and enhanced metabolic dysfunction. IL-13-treated bronchial epithelial cells and patient samples confirmed reduced mRNA and protein expression. Exploratory single-cell analysis revealed that HLA−DPA1/DPB1 were enriched in macrophages, specifically a Macro2 subset characterized by metabolic and stress-related functions—highlighting macrophage heterogeneity in innate immune regulation. Pseudotime trajectory suggested a shift from immune-activated toward metabolically stressed states. Cell−cell communication analysis identified epithelial cells as primary signal senders, with macrophages and dendritic cells as central receivers, and the MIF signaling axis as a key intercellular bridge.

**Conclusion:**

This multi-level integrated transcriptomic analysis identified HLA-DPA1 and HLA-DPB1 as candidate key genes in childhood asthma, and reveals their potential role in immune-metabolic dysregulation centered on macrophage functional heterogeneity. Our data are consistent with a potential role for these genes in immune-metabolic dysregulation centered on macrophage functional heterogeneity, although direct functional validation is required to establish causality. These findings provide new insights into innate immune circuits in childhood asthma and lay a foundation for potential molecular targets for future precision therapeutic strategies.

## Graphical Abstract

**Figure:**
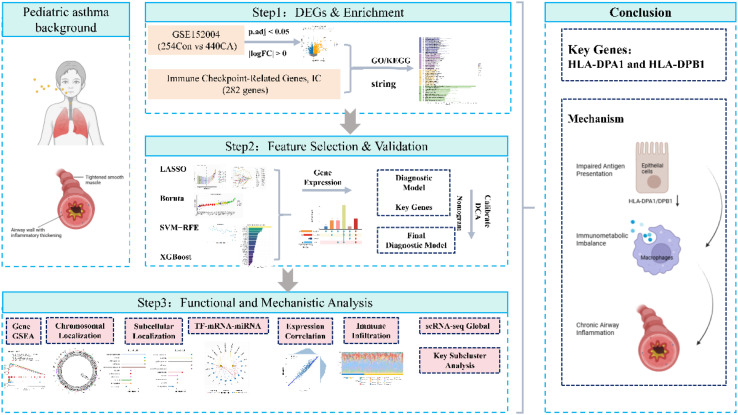

## Introduction

Asthma is a pervasive chronic respiratory disease affecting individuals from childhood through adulthood (1). It is clinically characterized by recurrent episodes of coughing, wheezing, chest tightness, and dyspnea, which are underpinned by significant airway remodeling and chronic inflammation (2, 3). The Global Burden of Disease (GBD) study estimated that, by 2021, there were approximately 95.72 million cases of Pediatric Asthma (PA) worldwide in the 0–14 age group, with an associated mortality rate of 0.41%. This high prevalence and disease impact represent a significant economic and healthcare burden for families and societies (4). PA is characterized by marked clinical heterogeneity and pathophysiological complexity, primarily shaped by gene–environment interactions (5). Inherited immune regulatory traits—particularly genetic predispositions in antigen presentation, cytokine signaling, and immune cell differentiation—play a critical role in disease persistence and airway remodeling (6, 7). The transition from childhood to adulthood asthma remains a poorly understood phase, often described as a ‘black box’ in the disease continuum. Many children with PA show limited response to existing therapies, leading to refractory or persistent disease—a major unmet clinical need. Thus, deeper mechanistic insights are urgently required to identify novel druggable pathways and enable more targeted and effective interventions.

The pathogenesis of PA involves a complex immunoinflammatory network, with dysregulatedimmune cell responses at its core. In allergic PA, a prominent T helper type 1/type 2 (Th1/Th2) imbalance leads to overactive Th2 responses and excessive production of IL-4, IL-5, and IL-13. These mediators, in turn, drive key pathological features including eosinophilic infiltration, elevated IgE production, and airway hyperresponsiveness (8, 9). In contrast, non-type-2 (T2-low) asthma encompasses neutrophilic or paucigranulocytic inflammation mediated Th1 and Th17 responses, which are often less responsive to corticosteroids. Recognizing these mechanistically distinct endotypes underscores the importance of dissecting immune heterogeneity in pediatric asthma (10, 11). Additionally, dysregulated macrophage polarization is recognized as a key contributor within the asthmatic immune milieu. Notably, macrophages polarized toward an M2 phenotype exacerbate disease pathology by releasing mediators such as YM1 protein, which actively promote airway inflammation and tissue remodeling (12). Innate immune activation also plays a significant role; Toll-like receptors (TLRs) recognize pathogen-associated molecular patterns (PAMPs) and critically regulate immune response magnitude and orientation (13). Despite technological advances that have enriched our understanding of PA immunology, critical knowledge gaps remain.

Immune checkpoints (ICs)are crucial regulators of immune responses, fine-tuning the intensity and duration of immune activation to prevent excessive tissue damage (14). Within the dysregulated immune milieu of asthma, ICs may act as decisive switches influencing disease onset, persistence, and severity. Investigating their role in PA could elucidate how genetic susceptibility translates into altered immune cell activity, offering novel perspectives for disease endotyping and personalized therapy (15). Among immune checkpoint-related molecules, HLA-DPA1 and HLA-DPB1 are candidate key genes encoding the DP subunits of human leukocyte antigen (HLA)-class II molecules, located within the HLA class II gene cluster on chromosome 6p21.32. These chains assemble into a heterodimer on antigen-presenting cells (such as macrophages, dendritic cells, B lymphocytes), where they present exogenous antigen peptides to activate CD4^+^ helper T cells, playing a central role in adaptive immune initiation and regulation. Genome-wide association studies (GWAS) have confirmed that the HLA class II region is a major susceptibility locus for asthma. Notably, the HLA-DPB1 rs9277534 variant influences antigen-binding affinity and immune response strength (16), and genetic variations in HLA-DPA1 have been associated with airway hyperresponsiveness and asthma severity (17). Recent evidence suggests that dysregulated expression of HLA-DPA1 and HLA-DPB1 may contribute to remodeling of the airway immune–metabolic microenvironment. However, their specific mechanisms in PA pathogenesis remain to be further elucidated.

Although accumulating evidence indicates that immune checkpoint molecules are involved in allergic and inflammatory diseases, systematic screening and functional characterization of specific immune checkpoint-related key genes in pediatric asthma remain limited. Previous studies have mostly focused on individual candidate molecules or specific immune pathways, leaving a gap in the comprehensive and unbiased identification of core immune checkpoint regulators in the airway epithelium of affected children.

To address this gap, this study integrates bioinformatics and machine learning approaches to systematically characterize the expression profiles and diagnostic value of HLA-DPA1 and HLA-DPB1 in childhood asthma based on bulk transcriptome and single-cell RNA sequencing data. Combined with *in vitro* experiments and patient sample validation, we further reveal their distribution patterns in immune cell subsets and the intercellular communication network through single-cell analysis, aiming to provide new insights into the immune-metabolic regulatory mechanisms of childhood asthma and establish a theoretical foundation for the development of precision therapeutic strategies.

## Materials and methods

### Data source

Transcriptomic datasets were acquired from the Gene Expression Omnibus (GEO) database, encompassing both bulk and single-cell RNA sequencing (scRNA-seq) data. Specifically, pediatric asthma-related datasets were retrieved using the keywords “asthma,” “pediatric,” and “gene expression.” Inclusion criteria required pediatric asthma (PA) and healthy control groups, and accessible expression matrices. Patients with other significant comorbidities, those receiving systemic medications that may significantly affect immune status, and studies lacking a control group were excluded. Specifically, two bulk transcriptomic datasets were utilized: the training set GSE152004 (GPL11154) contained high-throughput sequencing data from 440 PA and 254 control airway epithelium tissues. The validation set GSE65204 (GPL14550) contained microarray sequencing data from 36 PA and 33 control airway epithelium tissues. To provide cellular-level resolution, we also incorporated a scRNA-seq dataset, GSE254127 (GPL24676) comprised high-throughput transcriptome sequencing data from two airway samples. Additionally, from the literature (18), 282 genes related to immune checkpoint pathways (ICRGs) were obtained (**Supplementary Table 1**).

### Acquisition of candidate genes

In the GSE152004 training set, differential expression analysis between PA and control samples was performed using the “DESeq2” package (v 1.46.0) (19). To capture a comprehensive landscape of transcriptomic alterations and avoid the premature exclusion of biologically significant genes, differentially expressed genes (DEGs) were identified with a permissive thresholds of |log_2_Fold Change (FC)| > 0 and adjusted p-value (p.adj) < 0.05. Subsequently, a Venn diagram was constructed using the “VennDiagram” package (v 1.7.3) to identify the intersection between the obtained DEGs and the ICRGs (20). The overlapping genes were defined as candidate genes.

### Functional enrichment analysis and protein-protein interaction network construction

To elucidate the biological processes and functions associated with the candidate genes, Gene Ontology (GO) and Kyoto Encyclopedia of Genes and Genomes (KEGG) enrichment analyses were performed using the “clusterProfiler” package (v 4.15.1.1) (21). GO analysis encompasses three categories: biological processes (BP), cellular components (CC), and molecular functions (MF). The top 10 GO terms and KEGG pathways were then plotted with a significance threshold of adj.p < 0.05. To further elucidate protein-protein interactions among the candidate genes, a PPI network was constructed using the STRING database (https://cn.string-db.org/) with confidence > 0.15.

### Screening of candidate key genes

Feature selection was performed on the training set GSE152004 using four distinct machine learning algorithms, to ensure high robustness and minimize algorithm-specific bias. Support vector machine-recursive feature elimination (SVM-RFE) model was implemented with the “caret” package (v 6.0.94) (22)using 10-fold cross-validation; the optimal gene subset was determined as the one yielding the highest accuracy during recursive elimination. The Boruta algorithm was applied using the “Boruta” package (v 8.0.0) (23)to identify all relevant features by comparing original attributes with random shadow features. Irrelevant features were iteratively eliminated across up to 100 runs, with a significance threshold of p < 0.01. Additionally, least absolute shrinkage and selection operator (LASSO) regression analysis was conducted using the “glmnet” package (v 4.1.8) (24) with 10-fold cross-validation selected features at the minimum lambda value, applying L1 regularization to prevent overfitting. An XGBoost classification model was constructed using the “xgboost” package (v 1.7.8.1), with hyperparameter tuning via 10-fold cross-validation (nrounds = 100), retaining genes with importance score > 0. The model was trained with a fixed nrounds=100, and genes with an importance score greater than zero were considered candidate features (25). Finally, the UpSetR package (v 1.4.0) was utilized to identify the intersecting feature genes derived from the four machine learning algorithms, which were defined as candidate key genes (26).

Expression levels of these candidate key genes were validated in the independent dataset GSE65204. Genes consistently and significantly differentially expressed in both cohorts were defined as the final candidate key genes.

To independently validate the robustness of selected features, the same screening pipeline (differential expression followed by intersection with ICRGs and three machine learning algorithms: LASSO, SVM-RFE, and XGBoost) was applied to the validation cohort GSE65204, using a stricter DEG threshold (|log2FC| > 0.5, p < 0.05).

### Construction of the nomogram

To evaluate the diagnostic value of candidate key genes for PA, a nomogram model of the candidate key genes was constructed using the “rms” package (v 6.8.1) (27), based on the training set GSE152004, with PA status as the outcome event. Each key gene was assigned an individual point, and the sum of these scores corresponded to the total points, which was then used to predict the probability of developing PA. To further assess the diagnostic performance of the key gene-based diagnostic model, a calibration curve was plotted using the “rms” package (v 6.8.1). To evaluate the clinical utility of the diagnostic model, a decision curve was generated using the “rmda” package (v 1.6) (28), illustrating the net clinical benefit. Unlike traditional binary classification models, the nomogram provides an individualized predicted probability rather than categorizing patients into fixed risk groups based on a predefined cutoff value.

### Gene set enrichment analysis

To explore biological pathways associated with HLA-DPA1 and HLA-DPB1, we performed a single-gene GSEA. All genes were ranked by their Spearman correlation coefficients with each key gene’s expression across samples. This ranked list was then utilized for GSEA to identify biological pathways positively or negatively associated with the key gene. Subsequently, the “c2.cp.kegg_legacy.v2025.1.Hs.symbols.gmt” gene set from the Molecular Signatures Database (MSigDB, https://www.gsea-msigdb.org/gsea/msigdb/) was used as the reference. GSEA was then performed for each key gene using the “clusterProfiler” package (v 4.15.1.1), with |Normalized enrichment score (NES)| > 1 and p.adj < 0.05 considered statistically significant enrichment results. Pathways that were significantly co-enriched across candidate key genes were visualized. Finally, the “GSVA” package (v 1.46.0) was employed to evaluate the GSVA scores of these common pathways in the PA and control groups (29), and the Wilcoxon test was used to compare the pathway activity between the two groups.

### Immune cell infiltration analysis

The relative abundance levels of 22 immune cell types in each sample from the training set GSE152004 were quantified using the “CIBERSORT” package (v 0.1.0) (30, 31). Using the control group as a reference, the Wilcoxon test was employed to assess the differences in immune cell infiltration between the PA and control groups. Subsequently, the cor function in R was applied to analyze correlations among the differentially infiltrated immune cells and further to evaluate the associations between the candidate key genes and these immune cells (|correlation coefficient (cor)| > 0.3, p < 0.05).

### Gene localization and functional association analysis

Gene mapping is crucial for investigating gene structure, function, and interactions. Chromosomal distribution of candidate key genes was visualized using the RCircos package (v 1.2.2) (32), while mRNA subcellular localization was predicted via the GeneCards database (https://www.genecards.org/). To explore interactions among candidate key genes, Spearman correlation analysis was performed across all samples in the training set GSE152004, with thresholds set at |cor| > 0.3 and p < 0.05. Furthermore, to gain deeper insights into the functional relationships among candidate key genes, gene ontology semantic similarity assessment and expression correlation analysis were conducted.

### Cell culture and treatment

BEAS-2B (normal human bronchial epithelial) cells were purchased from ATCC and maintained in DMEM at 37 °C. Cells were routinely tested for mycoplasma contamination using PCR-based assays, and only mycoplasma-free cultures were used for experiments. To establish an *in vitro* cell model, BEAS-2B cells were treated with IL-13 (20 ng/mL) for 24 hours, a well-established asthma-inducing cytokine.

### Human samples

Asthma diagnosis was established in accordance with the Global Initiative for Asthma (GINA) guidelines (33). Children diagnosed with asthma, aged 6 to 15 years, were enrolled in the study. Exclusion criteria included the presence of any other respiratory diseases besides asthma. Age-matched healthy children were recruited as the normal control group. Peripheral blood mononuclear cells (PBMCs) were isolated from peripheral blood samples by density gradient centrifugation using Ficoll-Paque according to the manufacturer’s instructions. The study protocol was approved by the Medical Ethics Committee of the Seventh Affiliated Hospital of Sun Yat-sen University (Approval Number: SYSU-KY-2025-565-01). Written informed consent was obtained from all participants and/or their legal guardians.

### Reagents

Antibodies against HLA-DPA1 and HLA-DPB1 were purchased from Proteintech (Wuhan, China). The fluorescent-conjugated secondary antibodies anti-mouse and anti-rabbit IgG were from LI-COR Biotechnology (Nebraska).

### Peripheral blood mononuclear cell isolation from patient samples

PBMCs from PA patients were isolated using density gradient centrifugation for downstream assays. Briefly, peripheral blood samples were diluted 1:1 with phosphate-buffered saline (PBS). A 15 mL conical tube was prepared with an equal volume of lymphocyte separation medium. The diluted blood was carefully layered onto the separation medium without mixing. The tube was centrifuged at 1500 × g for 5 minutes at room temperature with the brake off. After centrifugation, the buffy coat layer containing PBMCs was carefully aspirated using a pipette and transferred to a new 15 mL tube pre-chilled to 4 °C. The collected cells were washed with an equal volume of ice-cold PBS and centrifuged. The cell pellet was then resuspended in an appropriate volume of red blood cell lysis buffer, mixed thoroughly, and incubated on ice for approximately 10 minutes. Following lysis, the suspension was centrifuged, and the pellet was washed twice with PBS to remove debris. The final PBMC pellet was either processed immediately or stored at -80 °C for future use.

### Western blotting analysis

Whole-cell lysates were prepared in radio immunoprecipitation assay (RIPA) buffer [1× PBS, 1% NP-40, 0.5% sodium deoxycholate, 0.1% sodium dodecyl sulfate (SDS)] supplemented with 10 mmol/L β-glycerophosphate, 1 mmol/L sodium orthovanadate, 10 mmol/L NaF, 1 mmol/L phenylmethylsulfonyl fluoride, and 1× Roche complete Mini protease inhibitor cocktail (Roche, Indianapolis, IN). Protein samples were separated by SDS-polyacrylamide gel electrophoresis and transferred to nitrocellulose membranes, which were then incubated with the primary antibodies. After incubation with appropriate secondary antibodies, the membranes were scanned by the Odyssey infrared imaging system (LI-COR, Lincoln, Nebraska).

### Real-time quantitative PCR

Total RNA was isolated from samples using TRIzol reagent (Invitrogen, Shanghai, China). Two micrograms of RNA were reversely transcribed into cDNA using the SuperScript III First-Strand Kit (Invitrogen, Shanghai, China) under the following conditions: 25 °C for 5 min (primer annealing), 50 °C for 45 min (reverse transcription), and 85 °C for 5 min (enzyme inactivation). cDNA (60 ng) was used for real-time quantitative PCR (qRT-PCR) with SYBR Premix Ex Taq (Takara, Dalian, China) using CFX96 Real-Time Detection System (Bio-Rad Laboratories, Hercules, PA). Thermal cycling parameters included: initial denaturation at 95 °C for 30 sec, followed by 40 cycles of 95 °C for 5 sec and 60 °C for 30 sec, with melt curve analysis (65–95 °C, 0.5 °C increments) to confirm primer specificity. Primers for specific genes for the amplification of cDNA were listed in **Supplementary Table 8**.

### Construction of molecular regulatory networks

For regulatory mechanism investigation, miRNAs targeting candidate key genes were predicted using three algorithms—Miranda, Microcosm, and diana_microT. Only miRNA–target pairs consistently identified by at least two algorithms were defined as key miRNAs for subsequent analysis. To further elucidate transcriptional regulation, high-confidence Transcription factor (TF)–mRNA interactions were retrieved from the DoRothEA database (sourced from ENCODE, ChEA, and other public repositories). TFs regulating the target genes were assigned confidence-based weights (A = 5 to E = 1) and regulatory directions (activation as “Up”, inhibition as “Down”). Finally, a multi-level regulatory network was constructed using the “ggraph” package (v 2.2.2) to illustrate the central role and regulatory patterns of candidate key genes within the network (34).

### Single-cell analysis

The GSE254127 dataset was subjected to quality control using the “Seurat” package (v 5.0.1) (35). The selection criteria were as follows: genes expressed in more than 3 cells, cells with gene counts between 500 and 6000, cells with less than 30% mitochondrial genes, and cells with total gene counts less than 40,000 and gene numbers greater than 500. Gene expression data were log-normalized using the NormalizeData function. Subsequently, the FindVariableFeatures function was then used to identify the top 2,000 highly variable genes (HVGs).

To assess the overall distribution of cells and identify potential outlier samples across all samples, we performed data scaling using the ScaleData function. Dimensionality reduction was then carried out by performing principal component analysis (PCA) on the top 2,000 HVGs, using the RunPCA function. Statistically significant principal components were determined with the JackStrawPlot function, and the top 30 principal components (PCs) were selected for all subsequent analyses. Cell clusters were identified using the Seurat package (v 5.0.1), specifically the FindNeighbors and FindClusters functions. Finally, the resulting clusters were visualized using uniform manifold approximation and projection (UMAP). The different clusters were annotated by finding marker genes through the references (36, 37).

### Subcluster analysis of key cells

Key cells were further isolated for subclustering. Dimensionality reduction, clustering, and re-annotation were performed using the top 20 PCs and the top 1,000 HVGs. Clustering was performed with the FindNeighbors and FindClusters functions, and the resolution parameter was set to 0.1 to obtain a conservative number of clusters. Marker genes for each cluster were identified using the FindAllMarkers function (parameters: min.pct = 0.3, logfc.threshold = 0.25, test.use = “wilcox”, and only.pos = TRUE). To elucidate the biological pathways and functional roles of key cellular subpopulations in PA, functional enrichment analysis was conducted on the differential cell clusters using the ReactomeGSA package (v 1.20.0) (38), with p.adj < 0.05 considered statistically significant enrichment results.

### Pseudo-time analysis

Cellular pseudotime trajectories were reconstructed using Monocle (v 2.34.0) to model the progression of PA (39). The CellDataSet was built from the Seurat object, and genes expressed in ≥ 10 cells were used. Ordering genes were defined as those with a q-value < 0.01 from a differential gene test. The final trajectory was inferred using the DDRTree method for dimensionality reduction.

### Cell communication analysis

Based on all samples from the GSE254127 dataset, cell-cell communication analysis among all cell types was performed using the CellChat package (v 2.1.2) (40). Potential ligand-receptor interactions were calculated to identify the signaling interactions between cell types.

### Statistical analysis

The R language (v 4.2.2) was utilized to perform bioinformatic analyses. Additionally, the Wilcoxon test was used in this study to assess differences between groups, with a significance threshold of p < 0.05.

## Results

### Identification of differentially expressed genes and immune checkpoint-related candidates

**Figure 1:**
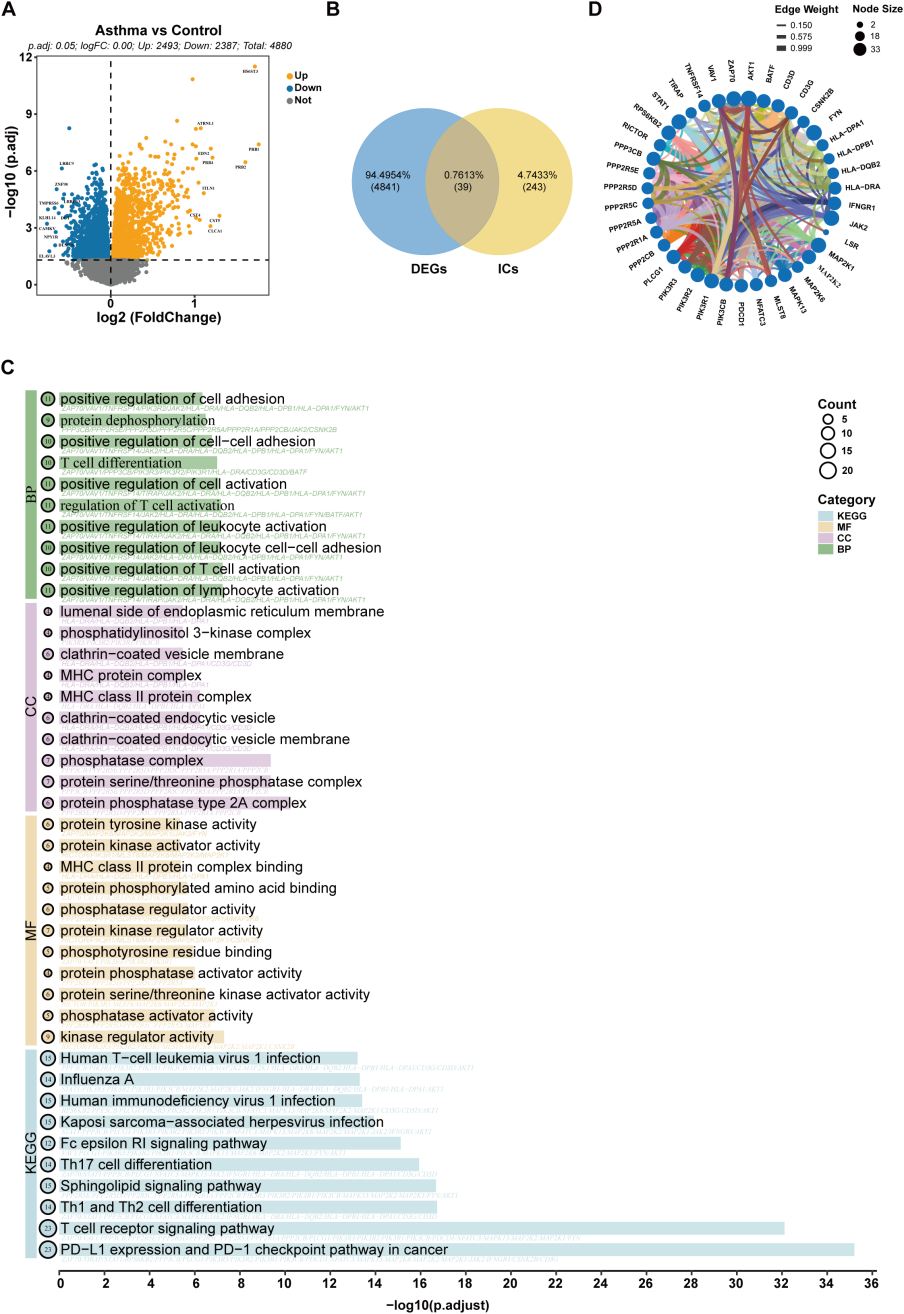
Identification of differentially expressed genes and immune-related candidates. **(A)** Volcano plot of the top 10 DEGs ranked by |log2FC|; **(B)** Venn diagram of the intersection between DEGs and ICRGs; **(C)** GO and KEGG enrichment results for candidate genes; **(D)** PPI network of the candidate genes.

To identify differentially expressed genes associated with immune checkpoint regulation in childhood asthma, we compared samples from pediatric asthma (PA) patients and healthy controls in the training set GSE152004. A total of 4,880 differentially expressed genes (DEGs) were identified, of which 2,493 were up-regulated and 2,387 were down-regulated in the PA group compared to controls (|log2FC| > 0, p.adj < 0.05) (**Figure 1A**; **Supplementary Table 2**). By intersecting these DEGs with immune checkpoint-related genes (ICRGs), we obtained 39 candidate genes (**Figure 1B**; **Supplementary Table 3**), suggesting that these genes may play pivotal roles in immune regulation in childhood asthma. Functional enrichment analysis revealed that these immune checkpoint-related candidate genes were significantly enriched in 478 GO terms, including 378 biological processes (e.g., positive regulation of lymphocyte activation, regulation of T cell activation), 47 cellular components (e.g., protein phosphatase type 2A complex), and 53 molecular functions (e.g., kinase regulator activity) (adj.p < 0.05) (**Figure 1C**; **Supplementary Table 4**), indicating that the candidate genes are primarily involved in the regulation of multiple immune responses. Furthermore, KEGG pathway analysis identified 154 significantly enriched pathways, mainly involving the PD-L1 expression and PD-1 checkpoint pathway, as well as the T cell receptor signaling pathway (p < 0.05) (**Figure 1C**; **Supplementary Table 4**), further supporting the potential importance of immune checkpoint pathways in the pathogenesis of childhood asthma. Subsequently, a protein-protein interaction (PPI) network was constructed based on the candidate genes to explore the interactions among these immune checkpoint-related genes (**Figure 1D**). The network revealed 380 interaction pairs among the 39 candidate genes (confidence > 0.15), suggesting close synergistic regulatory relationships that may collectively contribute to the immune regulatory network in childhood asthma. In summary, through differential expression analysis, functional enrichment, and PPI network construction, we systematically screened and preliminarily validated 39 immune checkpoint-related candidate genes, laying the foundation for the subsequent identification of candidate key genes.

### Consensus machine learning screening identifies HLA-DPA1 and HLA-DPB1 as candidate key genes

**Figure 2:**
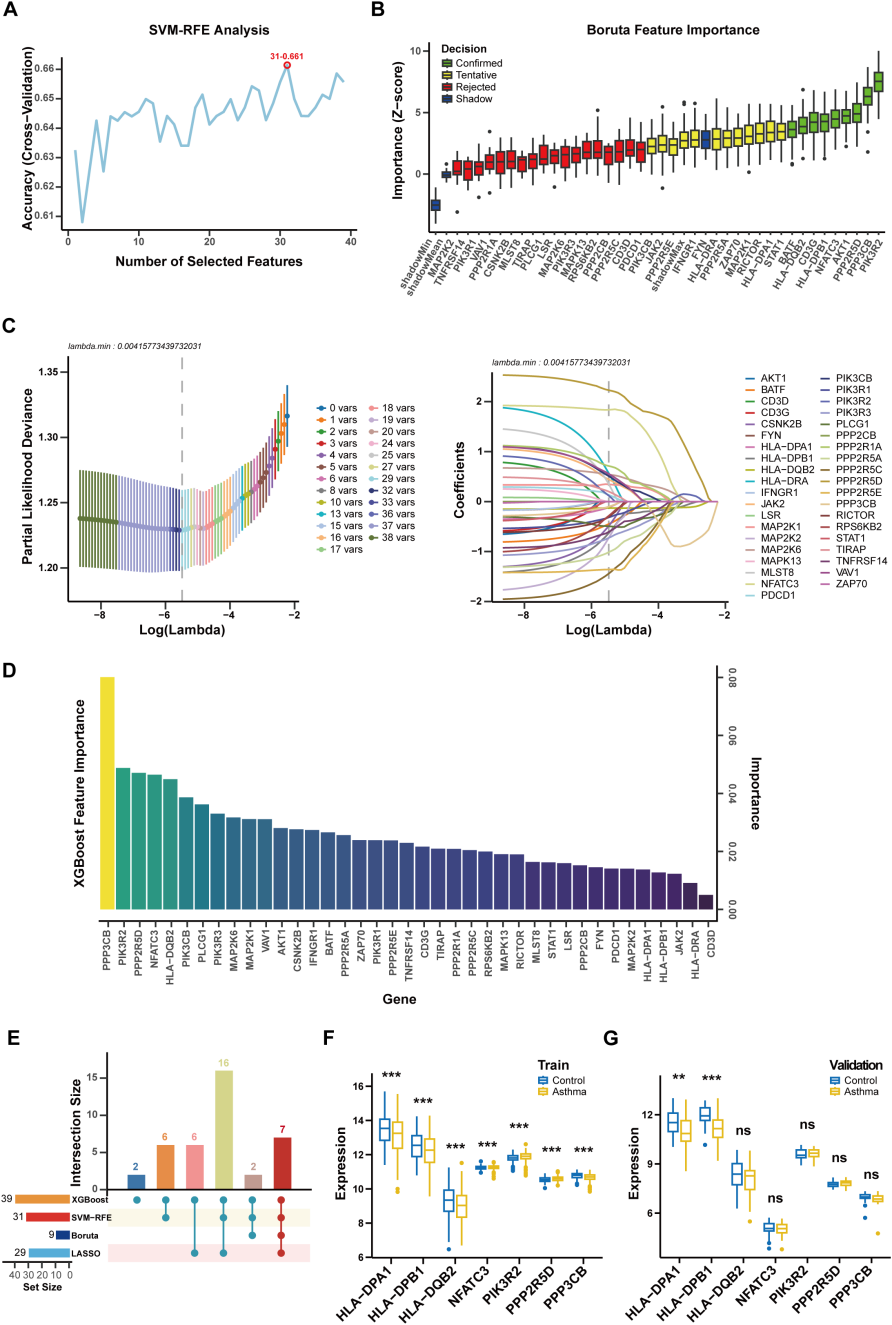
Consensus machine learning screening identifies HLA-DPA1 and HLA-DPB1 as candidate key genes. **(A)** Gene selection by SVM-RFE; **(B)** Feature importance and confirmation by Boruta; **(C)** Gene selection profile of the LASSO regression model, the coefficient trajectories are plotted through the log(lambda) series and the corresponding non-zero coefficients are selected based on the optimal lambda values (left); In the LASSO model, the optimal parameter λ is determined by 10-fold cross-validation, and the selection criterion is the minimum error plus a standard error, with the vertical line indicating the location of this standard error (right); **(D)** Feature importance ranking by XGBoost; **(E)** Intersection of candidate key genes identified by the four algorithms; **(F)** Analysis of gene expression levels in the training set; **(G)** Analysis of gene expression levels in the validation set. *p < 0.05, **p < 0.01, ***p < 0.001, ns ≥0.05.

To further identify key immune checkpoint-related genes in childhood asthma, we employed four machine learning algorithms for comprehensive analysis. SVM-RFE (Support Vector Machine-Recursive Feature Elimination) is a boundary-based supervised learning algorithm that iteratively removes features with the smallest contribution to classification, making it particularly suitable for feature selection in high-dimensional small-sample data. Using the SVM-RFE algorithm, we identified 31 signature genes from the 39 candidate genes, achieving an accuracy of 0.66 (**Figure 2A**; **Supplementary Table 5**), indicating that the candidate genes contain robust feature patterns capable of distinguishing between asthmatic and control states. Boruta is a full-correlation feature selection algorithm that compares original features with their randomized shadow counterparts to comprehensively identify all features relevant to the dependent variable, ensuring robust selection results. Boruta analysis confirmed 22 relevant genes and further refined a set of 9 core features (**Figure 2B**; **Supplementary Table 5**), suggesting that most candidate genes are indeed closely associated with the pathophysiological processes of childhood asthma. LASSO (Least Absolute Shrinkage and Selection Operator) regression is an embedded feature selection method that introduces an L1 penalty term to shrink some feature coefficients to zero, achieving variable selection while effectively preventing model overfitting, particularly suitable for high-dimensional data where the number of predictors exceeds the sample size. The LASSO regression model identified 29 genes at a lambda value of 0.0042 (log(lambda) = -5.48), near the optimal performance point (**Figure 2C**; **Supplementary Table 5**), demonstrating that these genes possess significant discriminative capacity for disease status within a penalized regression framework. XGBoost (eXtreme Gradient Boosting) is an ensemble learning algorithm based on decision trees that optimizes the loss function through gradient boosting and introduces regularization terms to enhance model generalization, effectively capturing complex nonlinear relationships among features. Similarly, the XGBoost algorithm selected 29 important feature genes (**Figure 2D**; **Supplementary Table 5**), further validating the importance of these genes in nonlinear models. The intersection of the genes selected by the four algorithms was visualized using a Venn diagram, yielding 7 common genes: HLA-DPA1, HLA-DPB1, HLA-DQB2, NFATC3, PIK3R2, PPP2R5D, and PPP3CB (**Figure 2E**). This highly consistent selection across different algorithms reflects a consensus in identifying key biological features and significantly reduces the risk of false positives associated with any single algorithm.

To rigorously address concerns about overfitting and dataset bias, we performed a completely independent analysis on the validation cohort GSE65204. In GSE65204, differential expression analysis (|log2FC| > 0.5, p < 0.05) identified 193 DEGs (89 up-regulated, 104 down-regulated) (**Supplementary Figures S1A, B**; **Supplementary Table 6**). Intersection of these DEGs with the ICRGs yielded 10 overlapping genes, including HLA-DPB1, CD3G, HLA-DRB1, HLA-DRB3, HLA-DRB4, CD3D, HLA-DQA1, HLA-DPA1, PDCD1, and CD86 (**Supplementary Figure S1C**). Three machine learning algorithms (LASSO, SVM-RFE, and XGBoost) were then applied independently to this 10-gene set. LASSO selected 8 genes (lambda.min = 0.0084) (**Supplementary Figure S2A**), SVM-RFE selected all 10 genes at maximal accuracy (**Supplementary Figure S2B**), and XGBoost ranked gene importance (**Supplementary Figure S2C**). The intersection of the three algorithm outputs yielded eight consensus genes, including HLA-DPB1, CD3G, HLA-DRB1, CD3D, HLA-DQA1, HLA-DPA1, PDCD1, and CD86 (**Supplementary Figure S2D**).

Among the intersecting genes, HLA-DPA1 and HLA-DPB1 were prioritized based on three criteria: their consistent and significant downregulation in both the training (GSE152004) and validation (GSE65204) datasets (p < 0.05, **Figures 2F, G**), their high connectivity in the protein-protein interaction network indicating hub roles in immune regulation, and their well-established function as MHC class II antigen-presenting molecules. Examination of expression distributions revealed no clear bimodal pattern, indicating the downregulation reflects overall transcriptional changes rather than allele-specific expression. Given their high PPI centrality, cross-validation across multiple machine learning algorithms, and independent external validation, HLA-DPA1 and HLA-DPB1 were definitively identified as candidate key genes, providing a solid foundation for subsequent mechanistic studies and diagnostic evaluation.

### Development and validation of a predictive nomogram based on candidate key genes

**Figure 3:**
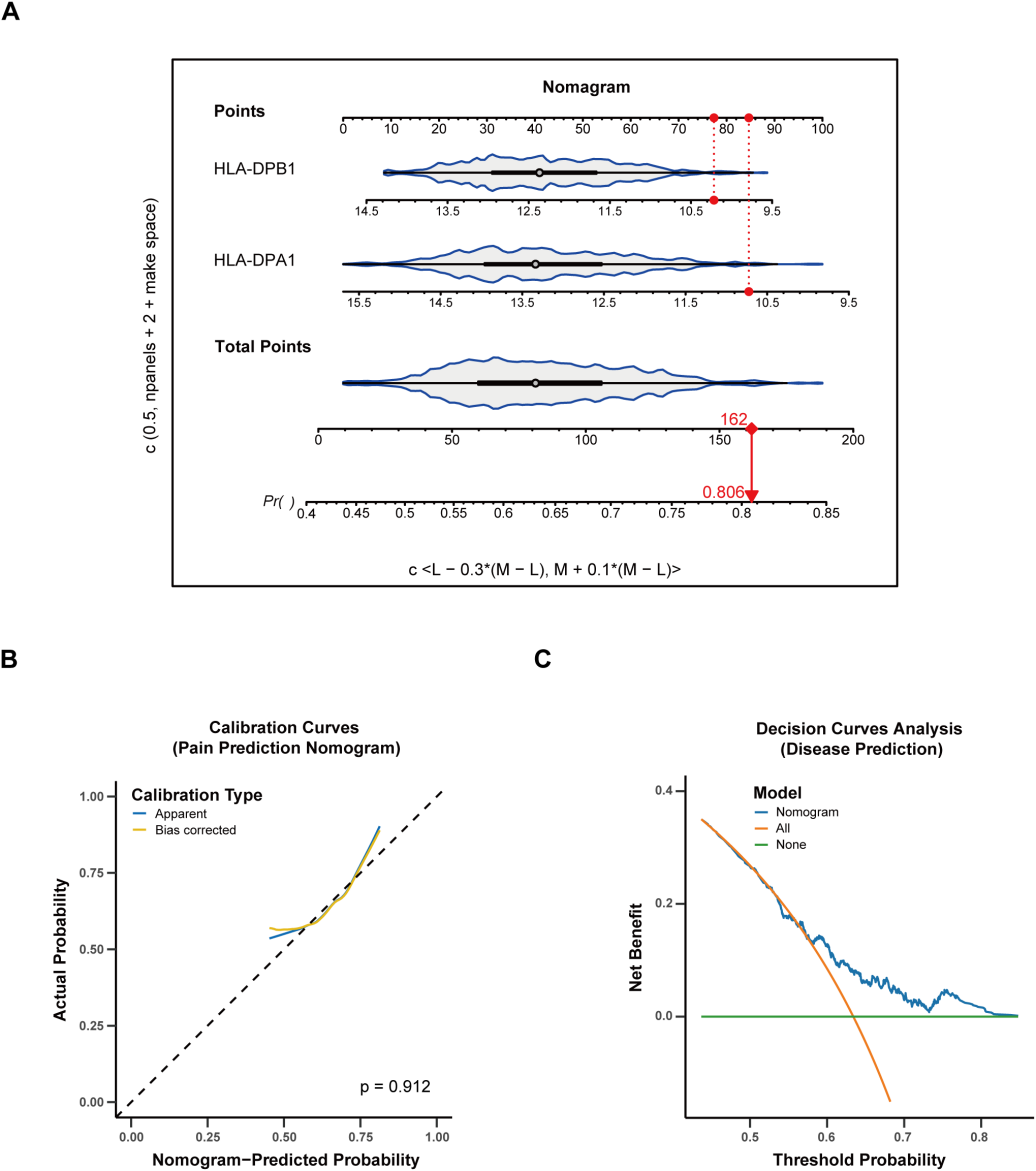
Development and validation of a predictive nomogram based on candidate key genes. **(A)** The nomogram for diagnosing PA; **(B)** The calibration curve of the nomogram; **(C)** Decision curves of the nomogram.

As a visualized risk prediction tool, nomograms transform complex regression models into intuitive graphical interfaces, enabling individualized quantification of disease probability by integrating multiple predictors, and thus hold broad application value in clinical decision support. Based on the candidate key genes HLA-DPA1 and HLA-DPB1 identified above, we constructed a nomogram model for predicting childhood asthma risk. The model demonstrated that the probability of childhood asthma gradually increased with increasing total points (**Figure 3A**), indicating a negative correlation between the expression levels of HLA-DPA1 and HLA-DPB1 and disease risk.

Calibration of the model was assessed using the Hosmer-Lemeshow goodness-of-fit test, which yielded a P-value of 0.912 (P > 0.05), indicating no significant discrepancy between the predicted probabilities and actual observed outcomes, suggesting that the nomogram possesses good calibration capability and accurately reflects true risk levels (**Figure 3B**). The clinical utility of the model was further evaluated through decision curve analysis, which assesses the value of predictive models in guiding clinical decisions by weighing the net benefits of true positives against false positives at different threshold probabilities. Our results showed that when the threshold probability ranged from 0.6 to 0.8—a clinically reasonable interval—the net benefit of the nomogram model was consistently higher than both the “treat all” and “treat none” strategies (**Figure 3C**), indicating that clinical decision-making guided by this model within this risk threshold range could yield clear clinical net benefit. Collectively, these results demonstrate that the nomogram model based on HLA-DPA1 and HLA-DPB1 not only exhibits good predictive accuracy and calibration but also possesses potential clinical applicability, providing a scientific basis for risk stratification and individualized intervention in childhood asthma.

### GSEA reveals dual association of candidate key genes with immune and metabolic pathways

**Figure 4:**
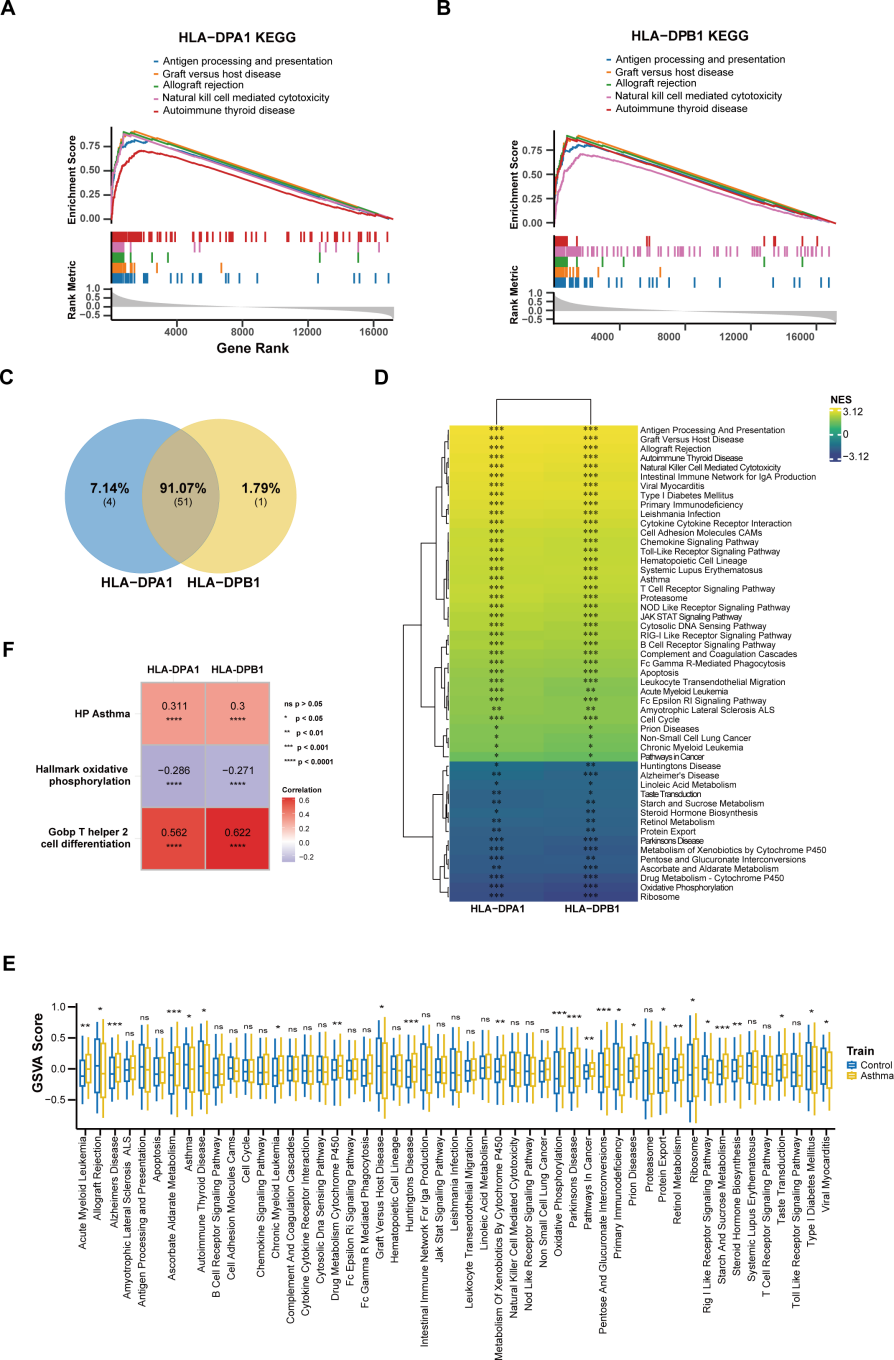
GSEA reveals dual association of candidate key genes with immune and metabolic pathways. **(A)** Top five enriched pathways for HLA-DPA1; **(B)** Top five enriched pathways for HLA-DPB1; **(C)** Biological pathways commonly enriched by the two candidate key genes; **(D)** Heatmap of NES scores for the biological pathways common to the two candidate key genes; **(E)** Box plot of co-enriched pathways; **(F)** GSVA correlations of HLA-DPA1/DPB1 with immune and metabolic pathways. *p < 0.05, **p < 0.01, ***p < 0.001, ns ≥0.05.

HLA-DPA1 and HLA-DPB1 encode the α and β chains of HLA class II DP molecules, which present exogenous antigens to activate T cells, serving as core regulators of adaptive immunity. GSEA revealed that HLA-DPA1 was significantly enriched in 55 pathways, and HLA-DPB1 in 52 pathways (|NES|>1, p.adj<0.05). Their top enriched pathways included antigen processing and presentation, graft-versus-host disease, allograft rejection, and natural killer cell-mediated cytotoxicity (**Figures 4A, B**; **Supplementary Table 7**). A total of 51 pathways were co-enriched (**Figure 4C**), indicating strong functional synergy. Notably, these shared pathways exhibited a biphasic pattern: positive correlations with immune pathways such as asthma and allograft rejection (NES>1), and negative correlations with metabolic and mitochondrial pathways including oxidative phosphorylation, ribosome, and protein export (NES<-1) (**Figures 4D, E**).

To substantiate genetic associations, we queried the GWAS Catalog for childhood asthma-related variants. Three independent signals were identified: rs111789468 and rs35449774 within HLA-DPA1, and rs3130169 mapping to both genes (**Table 1**). Additionally, GSVA showed that both genes were positively correlated with the asthma signature and Th2 differentiation (cor > 0.3, p < 0.05), and negatively correlated with oxidative phosphorylation (cor < -0.3, p < 0.05) (**Figure 4F**). Collectively, these findings reveal a dual role of HLA-DPA1 and HLA-DPB1 in pediatric asthma: their downregulation may attenuate antigen presentation while coinciding with enhanced metabolic dysfunction. This immune-metabolic dysregulation could jointly contribute to disease pathogenesis, offering new mechanistic insights.

**Table 1:** GWAS variants in HLA-DPA1/DPB1 for childhood asthma.

Variant	p-value	Mapped gene(s)	EFO trait	Study accession	Location (chr6)	PubMed ID
rs111789468	2×10^-^¹³	HLA-DPA1	childhood onset asthma	GCST007800	33,066,047	30929738
rs35449774	2×10^-^¹^6^	HLA-DPA1, HLA-DOA	childhood onset asthma	GCST009841	33,063,835	31669095
rs3130169	2×10^-8^	HLA-DPA1, HLA-DPB1	childhood onset asthma	GCST009841	33,080,325	31669095

All variants reached genome-wide significance (p < 5×10^-8^). Location coordinates are based on genome reference build GRCh37/hg19 as per the original GWAS accession.

### Characterization of immune microenvironment and its correlation with candidate key genes

**Figure 5:**
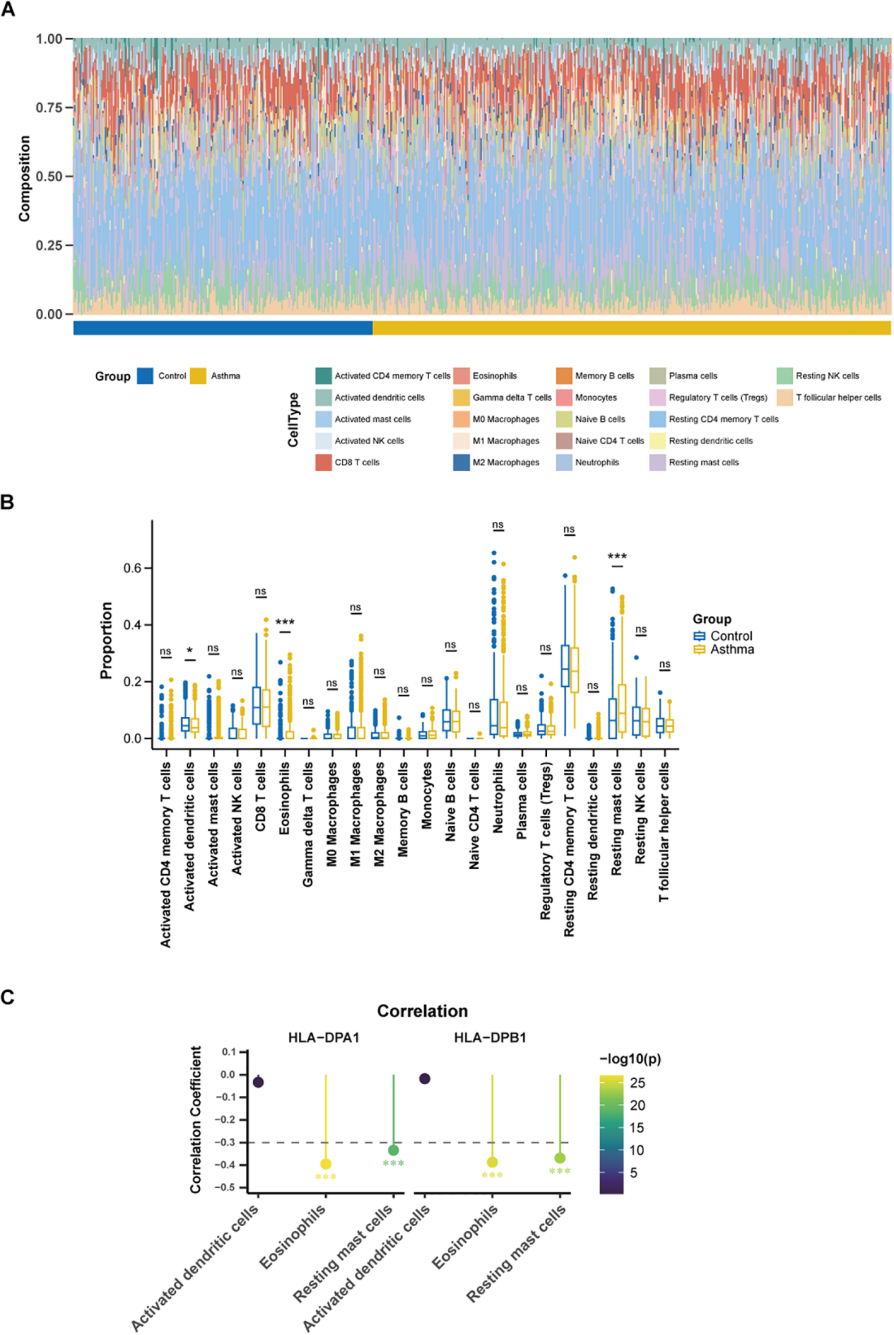
Characterization of immune microenvironment and its correlation with candidate key genes. **(A)** Comparison of 22 immune cell levels between PA and controls in GSE152004; **(B)** Box plot illustrating the differences in immune cell infiltration between PA and controls; **(C)** Correlation between the differentially infiltrated immune cells and candidate key genes. Note: Asterisks represent statistical significance: *p < 0.05, **p < 0.01, ***p < 0.001.

To further explore the role of HLA-DPA1 and HLA-DPB1 in the immune microenvironment of childhood asthma, we first evaluated the enrichment patterns of various immune cells through immune infiltration analysis. The results showed that activated dendritic cells and CD8 T cells exhibited relatively high enrichment scores in both the childhood asthma and control groups (**Figure 5A**), suggesting that these two cell types may serve as fundamental components of airway immune homeostasis. In the comparison between groups, only eosinophils and resting mast cells were significantly upregulated in the childhood asthma group, whereas activated dendritic cells were significantly downregulated (p < 0.05) (**Figure 5B**), indicating that the immune microenvironment in childhood asthma is characterized by an imbalance featuring enhanced infiltration of Th2-type inflammatory cells and suppressed antigen-presenting cell function. Further correlation analysis revealed that the expression levels of HLA-DPA1 and HLA-DPB1 were significantly negatively correlated with the abundance of eosinophils and resting mast cells (cor < -0.3, p < 0.05) (**Figure 5C**). This finding suggests that the downregulation of HLA-DPA1 and HLA-DPB1 may be closely associated with the recruitment or activation of Th2-type inflammatory cells, and these two genes may play synergistic roles in regulating asthma-related immune inflammatory responses. In summary, HLA-DPA1 and HLA-DPB1 are not only downregulated as candidate key genes in childhood asthma, but their expression changes are also closely related to the infiltration patterns of specific immune cells, further supporting their important status in the immune regulatory network of asthma.

### Genomic characterization and functional profiling of candidate key genes

Building upon the identification of HLA-DPA1 and HLA-DPB1 as candidate key genes in childhood asthma, we further systematically characterized the fundamental features of these two genes from multiple dimensions, including genomic localization, subcellular distribution, and functional annotation, to comprehensively understand their biological basis and potential roles in asthma pathogenesis. Both HLA-DPA1 and HLA-DPB1 are located within the MHC class II region on chromosome 6, exhibiting close spatial proximity that suggests a potential synergistic relationship at both the genetic and functional levels (**Supplementary Figure S3A**). In silico subcellular localization analysis revealed that HLA-DPA1 and HLA-DPB1 are localized to the cell membrane and extracellular region, with additional distribution observed in lysosomes, the endoplasmic reticulum, and the Golgi apparatus. This multi-compartmental localization pattern is highly consistent with their core functions in the antigen processing and presentation pathway (**Supplementary Figure S3B**). GO semantic similarity analysis demonstrated moderate to high consistency in the functional annotations of these two genes (**Supplementary Figure S3C**), further supporting their synergistic roles in biological processes. Gene correlation analysis revealed a significant positive correlation between their expression levels (cor = 0.936, p < 0.001) (**Supplementary Figure S3D**), suggesting the existence of common regulatory mechanisms at the transcriptional level. In summary, HLA-DPA1 and HLA-DPB1 not only exhibit close genomic proximity but also demonstrate high consistency in subcellular distribution, functional annotation, and expression regulation, further confirming their role as functionally synergistic units within the immune regulatory network of childhood asthma.

### Dissecting the multilayer transcriptional and post-transcriptional regulation of candidate key genes

Having established the genomic characteristics of HLA-DPA1 and HLA-DPB1 and their central role in the immune regulatory network, we further investigated the regulatory mechanisms of these two genes at both the transcriptional and post-transcriptional levels, aiming to elucidate the potential upstream regulatory factors underlying their dysregulated expression in childhood asthma. Through bioinformatic prediction, we constructed corresponding miRNA-mRNA regulatory networks. Analysis revealed that HLA-DPA1 is targeted by 4 miRNAs, while HLA-DPB1 is targeted by 2 miRNAs (**Supplementary Figure S4A**), based on which we constructed the corresponding mRNA-miRNA regulatory networks (**Supplementary Figure S4B**). Further analysis identified 9 and 8 transcription factors targeting HLA-DPA1 and HLA-DPB1, respectively. Based on these predictions, we constructed and visualized a transcription factor-mRNA regulatory network (**Supplementary Figure S4C**) as well as an integrated transcription factor-mRNA-miRNA triple regulatory network (**Supplementary Figure S4D**). The construction of this multi-layered regulatory network revealed that HLA-DPA1 and HLA-DPB1 may be subject to complex synergistic regulation by transcription factors and miRNA-mediated post-transcriptional modifications, providing potential upstream regulatory mechanisms underlying their downregulation in childhood asthma. These findings provide evidence for further experimental validation and contribute to a deeper understanding of the regulatory mechanisms of HLA-DPA1 and HLA-DPB1 in asthma pathogenesis.

### Experimental validation of key gene expression

**Figure 6:**
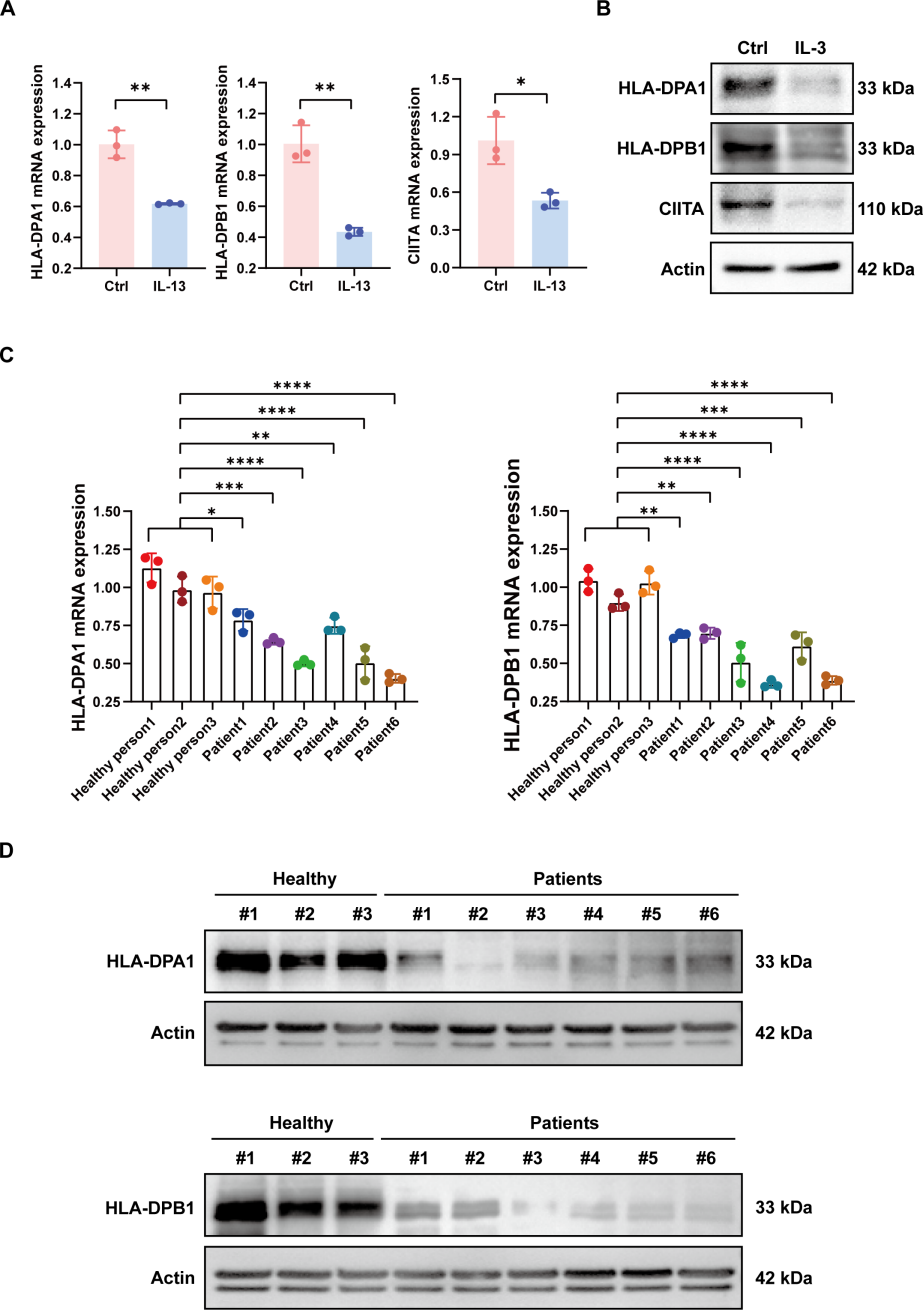
Experimental validation of HLA-DPA1 and HLA-DPB1 downregulation. **(A)** BEAS-2B human bronchial epithelial cells were treated with IL-13 (20 ng/mL) for 24 h to establish an *in vitro* asthma cell model. Quantitative real-time PCR analysis showed that the mRNA expression levels of HLA-DPA1, HLA-DPB1 and CIITA were significantly decreased in the IL-13-treated group compared with the control group. **(B)** BEAS-2B human bronchial epithelial cells were treated with IL-13 (20 ng/mL) for 24 h to establish an *in vitro* asthma cell model. Western blot analysis showed that the protein expression levels of HLA-DPA1, HLA-DPB1 and CIITA were significantly decreased in the IL-13-treated group compared with the control group. **(C)** PBMCs were collected from children with asthma and healthy controls. Quantitative qPCR analysis revealed that the mRNA expression levels of HLA-DPA1 and HLA-DPB1 were significantly lower in PBMCs from the asthma group compared with the healthy control group. **(D)** Western blot analysis was performed to assess the protein expression levels of HLA-DPA1 and HLA-DPB1 in PBMCs. The results demonstrated that the protein levels of both genes were markedly downregulated in the asthma group compared with the healthy control group. GAPDH was used as a loading control. *P<0.05; **P<0.01; ***P<0.001; ****P<0.0001, Student’s t-test, one-way ANOVA, *post hoc* comparisons, Tukey’s test.

To validate the expression characteristics of HLA-DPA1 and HLA-DPB1 in childhood asthma through both *in vitro* models and clinical samples, we first established an *in vitro* asthma cell model. IL-13 is a key effector cytokine secreted by Th2 cells and has been widely demonstrated to induce asthma-related pathophysiological changes, including airway epithelial barrier dysfunction, mucus hypersecretion, and inflammatory responses (41). Therefore, it is recognized as a well-established inducing factor for simulating the asthma microenvironment *in vitro*. Accordingly, we treated BEAS-2B human bronchial epithelial cells with IL-13 (20 ng/mL) for 24 hours to construct an *in vitro* asthma cell model. Quantitative real-time PCR analysis revealed that, compared with the control group, the mRNA expression levels of HLA-DPA1 and HLA-DPB1 were significantly decreased in IL-13-treated BEAS-2B cells, suggesting that these two genes are suppressed in asthma-related airway epithelial cell responses (**Figure 6A**).

On this basis, we further collected peripheral blood mononuclear cells (PBMCs) from children with asthma and healthy controls for clinical sample validation. Previous studies have shown that the airway epithelium, as the initiation site of the asthmatic immune response, exhibits immune status changes that can be reflected systemically through circulating immune cells, indicating close interaction and signal communication between the local airway and the systemic immune system (42–44). Multiple studies have also confirmed that immune-related molecules display consistent expression patterns in both the local airway and the systemic immune system (12, 45–49). Therefore, gene expression changes in PBMCs can serve as an important complement to the immune regulatory characteristics of the airway epithelium. Airway epithelium is the direct site of local inflammation in asthma, whereas PBMCs reflect the systemic circulating immune status. The two differ fundamentally in cellular composition, microenvironment, and functional aspects. Therefore, the validation results from PBMCs in this study should be considered as indirect support for the transcriptomic findings from airway epithelium, rather than as a strict substitute for validation.

Quantitative real-time PCR analysis confirmed that, compared with the healthy control group, the mRNA expression levels of HLA-DPA1 and HLA-DPB1 were significantly reduced in PBMCs from asthmatic children (**Figure 6B**). Consistent with this finding, Western blot analysis further demonstrated that the protein levels of HLA-DPA1 and HLA-DPB1 were also markedly downregulated in the asthmatic group (**Figure 6C**).

In addition, to explore which upstream transcriptional regulatory node may be responsible for the downregulation of HLA-DPA1/DPB1, we assessed the expression levels of a key transcriptional regulator of MHC class II molecules—specifically CIITA (class II major histocompatibility complex transactivator). The results showed that in the simulated asthma model, both mRNA and protein levels of CIITA were significantly downregulated compared with the normal control group, suggesting that CIITA, a critical transcriptional regulator of MHC class II molecules, likely mediates the downregulation of HLA-DPA1/DPB1 in the asthma model (**Figures 6A, B**).

The results of this dual validation, encompassing both the *in vitro* cell model and clinical samples, demonstrate that HLA-DPA1 and HLA-DPB1 exhibit a consistent downregulation trend, not only in bronchial epithelial cells simulating the asthma microenvironment but also in PBMCs reflecting the systemic immune status of affected children. This finding further confirms the pivotal role of these two genes in childhood asthma and suggests that their dysregulated expression may represent a common feature of immune dysfunction in asthma, providing a reliable experimental basis for subsequent functional mechanism studies.

### Construction of a single-cell atlas of the pediatric asthma

**Figure 7:**
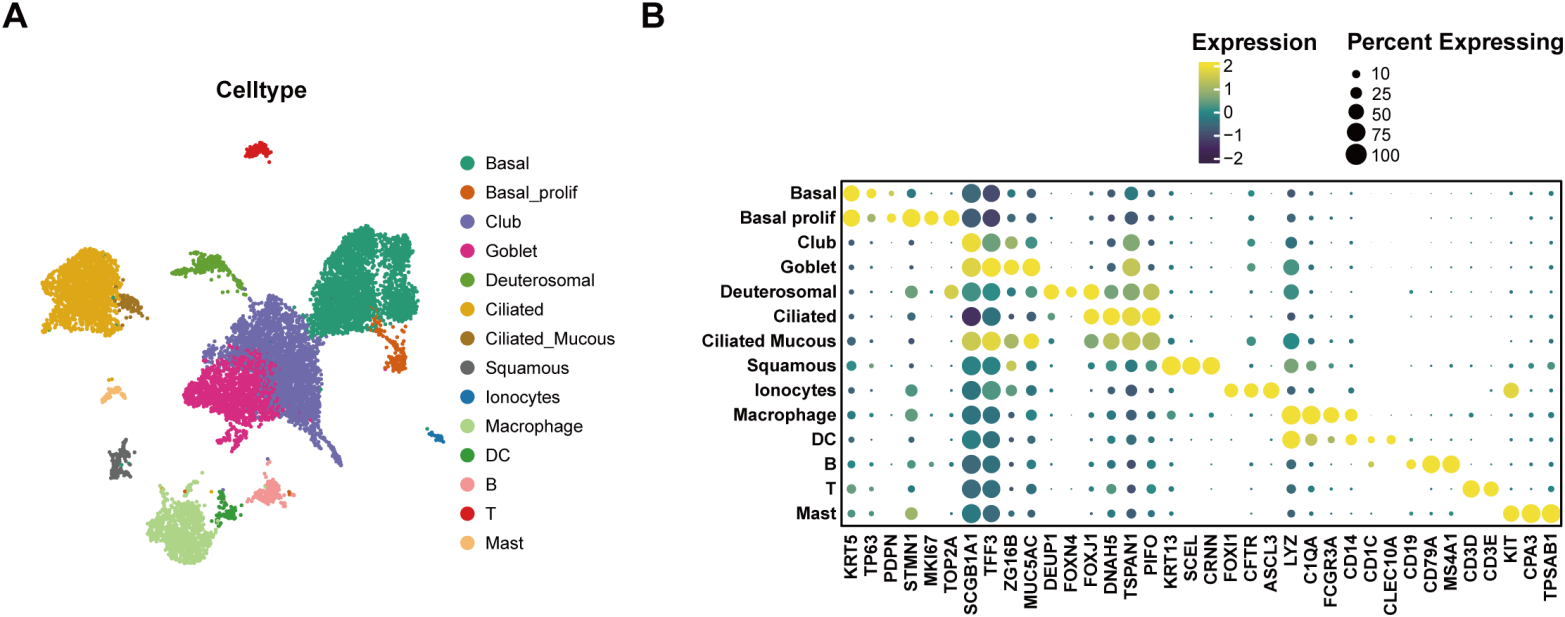
Construction of a single-cell atlas of the pediatric asthma. **(A)** Dimensionality reduction using the UMAP algorithm applied to the top 30 PCs, successfully classifying 14 cell clusters; **(B)** Expression levels of marker genes for each cell cluster.

Building upon the identification of HLA-DPA1 and HLA-DPB1 as candidate key genes in childhood asthma, we further analyzed single-cell RNA sequencing data from the airway epithelium of pediatric asthma patients to achieve a more refined resolution of the cellular origins and expression distribution patterns of these genes within the complex tissue microenvironment. Single-cell transcriptome sequencing enables the revelation of gene expression heterogeneity at single-cell resolution, overcoming the limitation of bulk sequencing that loses critical information due to cell type mixing, and facilitates elucidation of the roles of candidate key genes within specific cellular subpopulations. In the GSE254127 dataset, low-quality cells were first filtered out (**Supplementary Figure S5A**). Gene variance analysis was performed on the remaining high-quality cells, identifying 2,000 highly variable genes (**Supplementary Figure S5B**). Principal component analysis was applied to the two single-cell samples, and the top 30 principal components were selected for downstream analysis (**Supplementary Figure S5C**). These components were then used to cluster the cells into 14 distinct populations via the UMAP algorithm (**Figure 7A**). The expression of key marker genes across these cell types was visualized using a bubble plot (**Figure 7B**), and cellular subpopulations were annotated based on canonical marker genes (e.g., epithelial cells, immune cells). The above analysis successfully constructed a single-cell transcriptomic atlas of the airway epithelium in childhood asthma, laying the foundation for subsequent investigations into the expression patterns and functions of HLA-DPA1 and HLA-DPB1 within specific cellular subpopulations.

### Identification of functionally distinct macrophage subsets and pathway signatures

**Figure 8:**
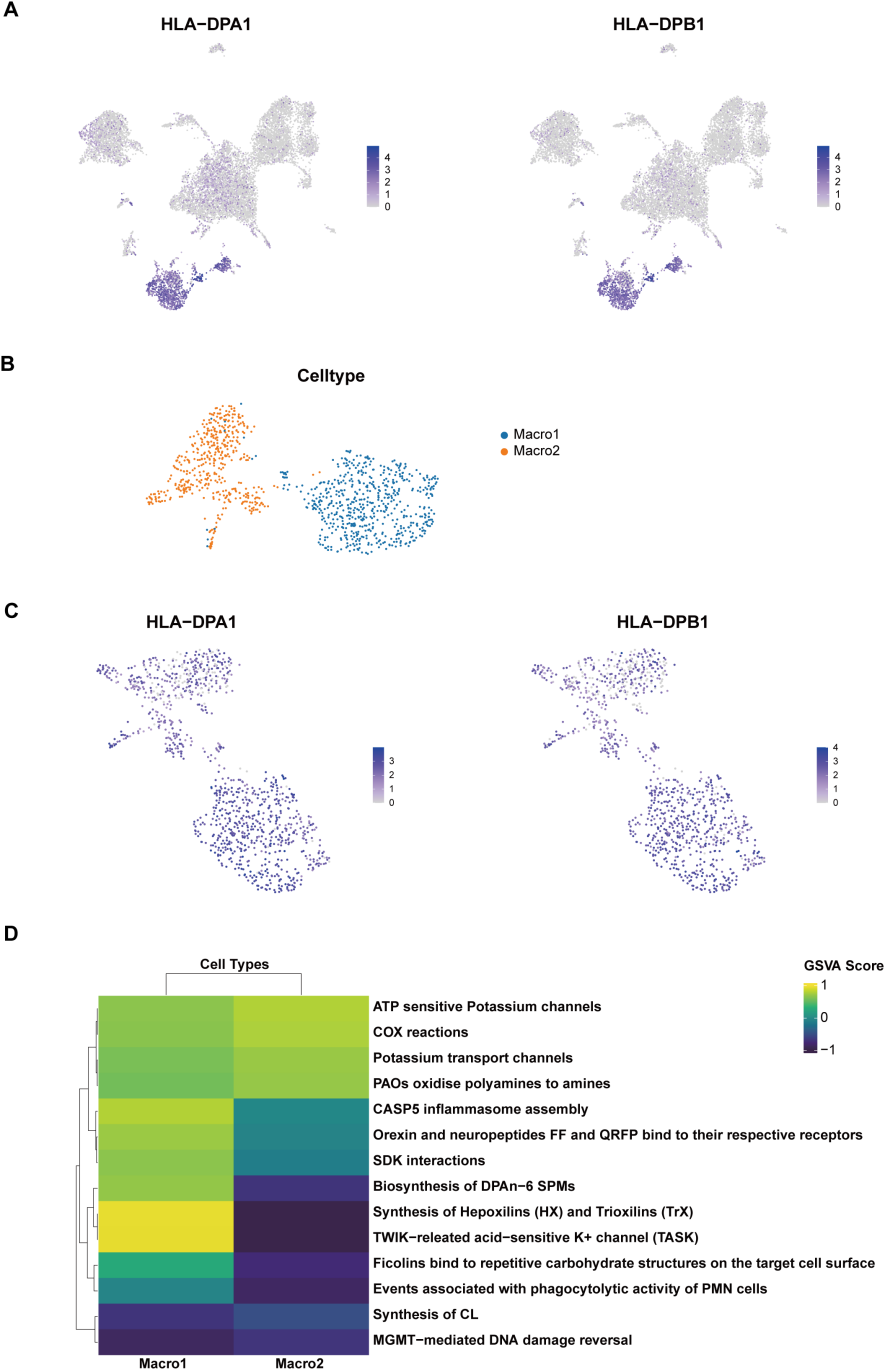
Identification of functionally distinct macrophage subsets and pathway signatures. **(A)** Expression of HLA-DPA1 and HLA-DPB1 across cell clusters; **(B)** Subclustering of macrophages yielding Macro1 and Macro2; **(C)** Expression of HLA-DPA1 and HLA-DPB1 in Macro1 and Macro2; **(D)** Heatmap showing significantly enriched pathways in key cell types.

Based on the construction of the single-cell atlas described above, we observed that HLA-DPA1 and HLA-DPB1 were significantly enriched in macrophages, dendritic cells, and B cells in pediatric asthma patients (**Figure 8A**). Macrophages, as core regulatory cells of the airway immune microenvironment, play a dual role in both initiating and resolving inflammation in asthma pathogenesis, and previous studies have confirmed their important role in pediatric asthma (50). However, macrophages exhibit high functional plasticity and heterogeneity, and traditional bulk sequencing is limited in its ability to distinguish distinct functional subsets during disease progression. Therefore, we performed an in-depth analysis of macrophages to investigate their cellular heterogeneity and potential functional specialization in pediatric asthma.

Subcluster analysis of macrophages was conducted using the top 20 principal components and the top 1,000 highly variable genes, with a clustering resolution of 0.1. This process resolved two computationally derived subsets, which we designate descriptively as Macro1 and Macro2 (These designations are dataset-specific). Notably, compared with Macro1, both candidate key genes HLA-DPA1 and HLA-DPB1 exhibited higher expression levels in the Macro2 subset (**Figures 8B, C**), suggesting that these genes may play more prominent roles in the functional specialization of Macro2. Functional enrichment analysis revealed distinct pathway signatures between these two subsets. Macro1 was primarily enriched in pathways related to immune recognition and inflammatory regulation, including ficolin-mediated pathogen recognition, polymorphonuclear cell-related phagocytic events, and specialized pro-resolving mediator biosynthesis (p.adj < 0.05), whereas Macro2 was significantly enriched in metabolic and stress-related pathways (p.adj < 0.05) (**Figure 8D**), including ATP-sensitive potassium channels and TWIK-related potassium channels, suggesting its potential role in regulating cellular excitability and immune activation thresholds through ion homeostasis. These functional annotations are based on ReactomeGSA and serve to characterize the clusters rather than to assign fixed biological identities.

These results indicate that in the airway epithelial microenvironment of pediatric asthma, macrophages exist as at least two functionally specialized subsets: Macro1 is characterized by immune recognition and inflammatory regulation, whereas Macro2 is oriented toward metabolic and stress responses. The higher expression of HLA-DPA1 and HLA-DPB1 in the Macro2 subset suggests that they may serve as important regulators of metabolism-associated macrophage function, participating in the immune-metabolic network regulation of asthma by influencing cellular metabolic status. This finding not only reveals the selective enrichment characteristics of candidate key genes in specific macrophage subpopulations but also provides new insights into the role of macrophage functional heterogeneity in the pathogenesis of pediatric asthma.

### Exploratory pseudotime and cell-cell communication analysis

**Figure 9:**
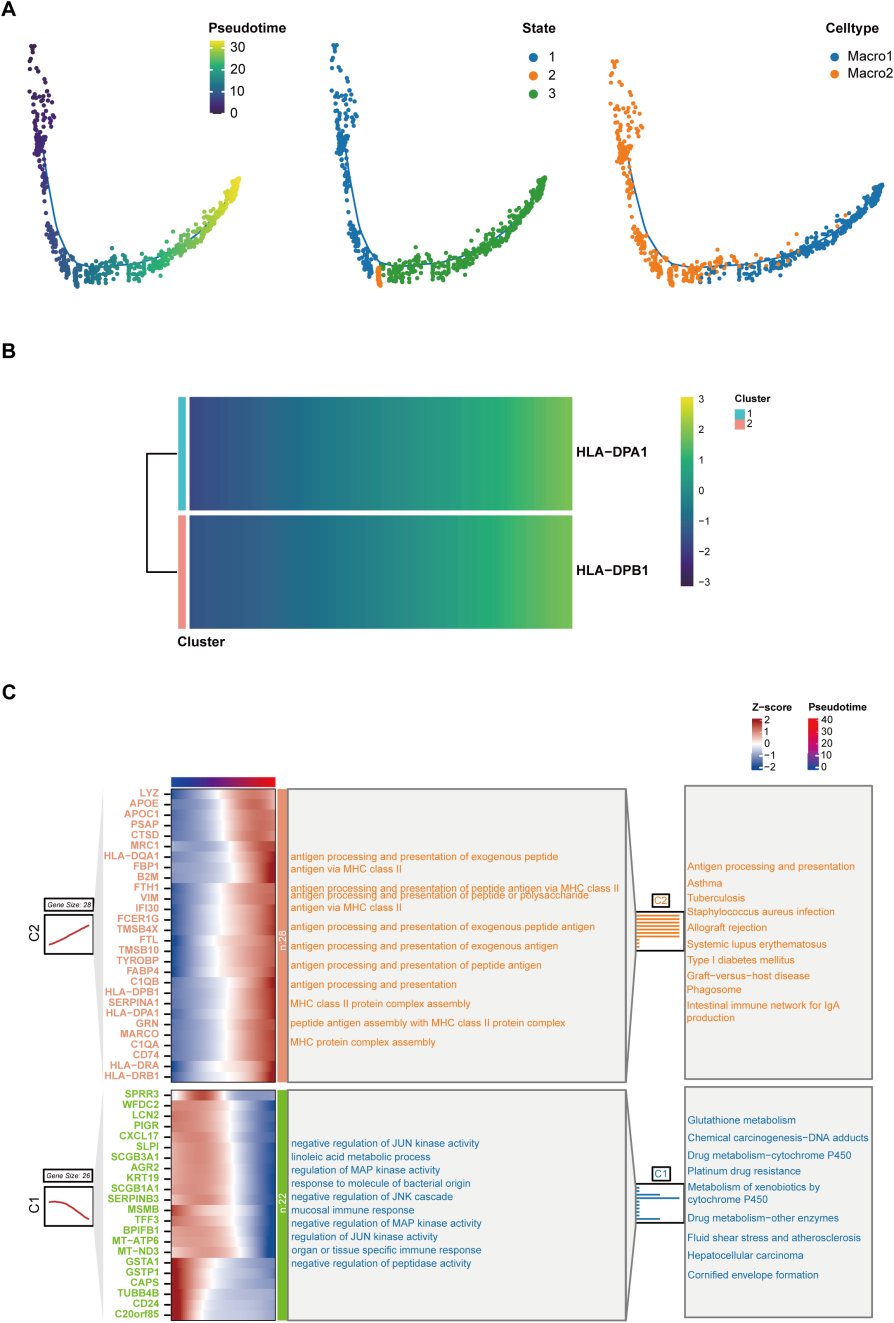
Developmental trajectory and functional bifurcation into metabolic-stress and immune-activated phenotypes. **(A)** Pseudotemporal trajectory analysis of Macro1 and Macro2; **(B)** Subclustering of macrophages based on HLA-DPA1/DPB1 expression; **(C)** Functional enrichment profiles of Clusters C1 and C2.

Following the identification of the functionally specialized macrophage subsets Macro1 and Macro2, we performed pseudotime analysis using the Monocle2 algorithm to further investigate the role of HLA-DPA1 and HLA-DPB1 in the dynamic functional transition of macrophages. Pseudotime analysis enables the inference of cellular state transitions along continuous developmental trajectories based on gene expression similarities, facilitating the elucidation of potential developmental relationships between macrophage subsets and the dynamic expression patterns of HLA-DPA1 and HLA-DPB1 therein. The trajectory analysis revealed that Macro1 and Macro2 were distributed along two distinct branches (**Figure 9A**). Due to the limited sample size (n=2), this trajectory should be viewed as a computational exercise rather than a validated developmental transition. The inferred branches may reflect inter-sample or technical variation rather than genuine biological states. Mapping the expression patterns of HLA-DPA1 and HLA-DPB1 onto the pseudotime trajectory revealed relatively higher expression levels of these two genes at later stages of the trajectory (**Supplementary Figure S6**), indicating that they may play important roles during the late phases of macrophage functional maturation or specialization. Based on the expression levels of HLA-DPA1 and HLA-DPB1, the cells were further divided into two subpopulations, designated Cluster 1 (C1) and Cluster 2 (C2) (**Figure 9B**). Functional enrichment analysis showed that C1 was primarily enriched in pathways related to immune response, signal transduction, and metabolic or stress-related pathways, whereas C2 was mainly enriched in MHC class II-related antigen presentation and immune activation pathways (**Figure 9C**), further confirming that the expression levels of HLA-DPA1 and HLA-DPB1 are closely associated with the immune-metabolic functional status of macrophages.

**Figure 10:**
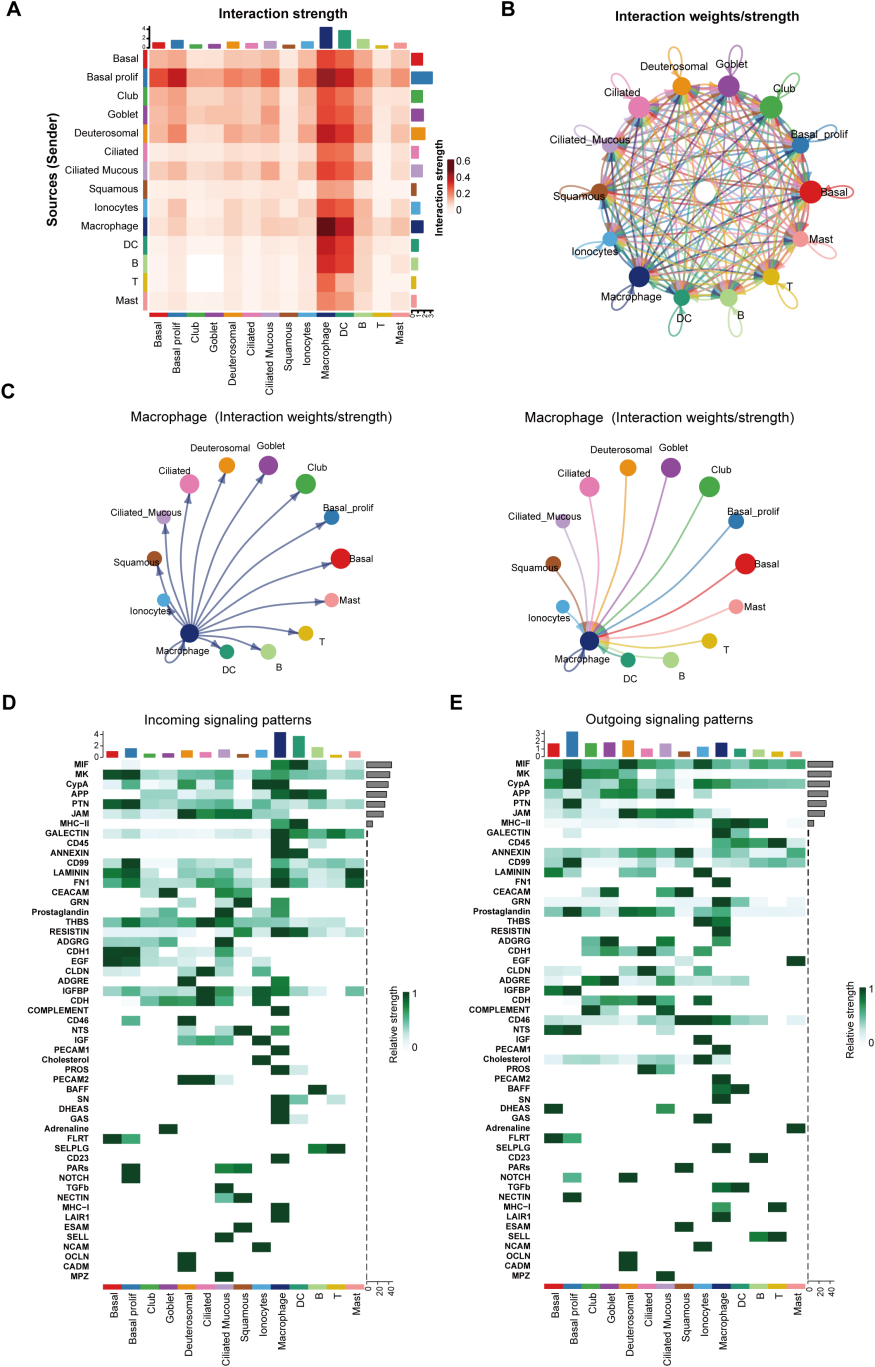
Cell-cell communication analysis reveals a dominant epithelium-to-innate immunity signaling pattern. **(A)** Heatmap of ligand-receptor interaction counts across cell types; **(B)** Differential roles of cell types as signal senders or receivers; **(C)** Specific interaction network between macrophages and Basal epithelial cells; **(D)** Incoming signaling patterns across cell types; **(E)** Outgoing signaling patterns across cell types.

**Figure 11:**
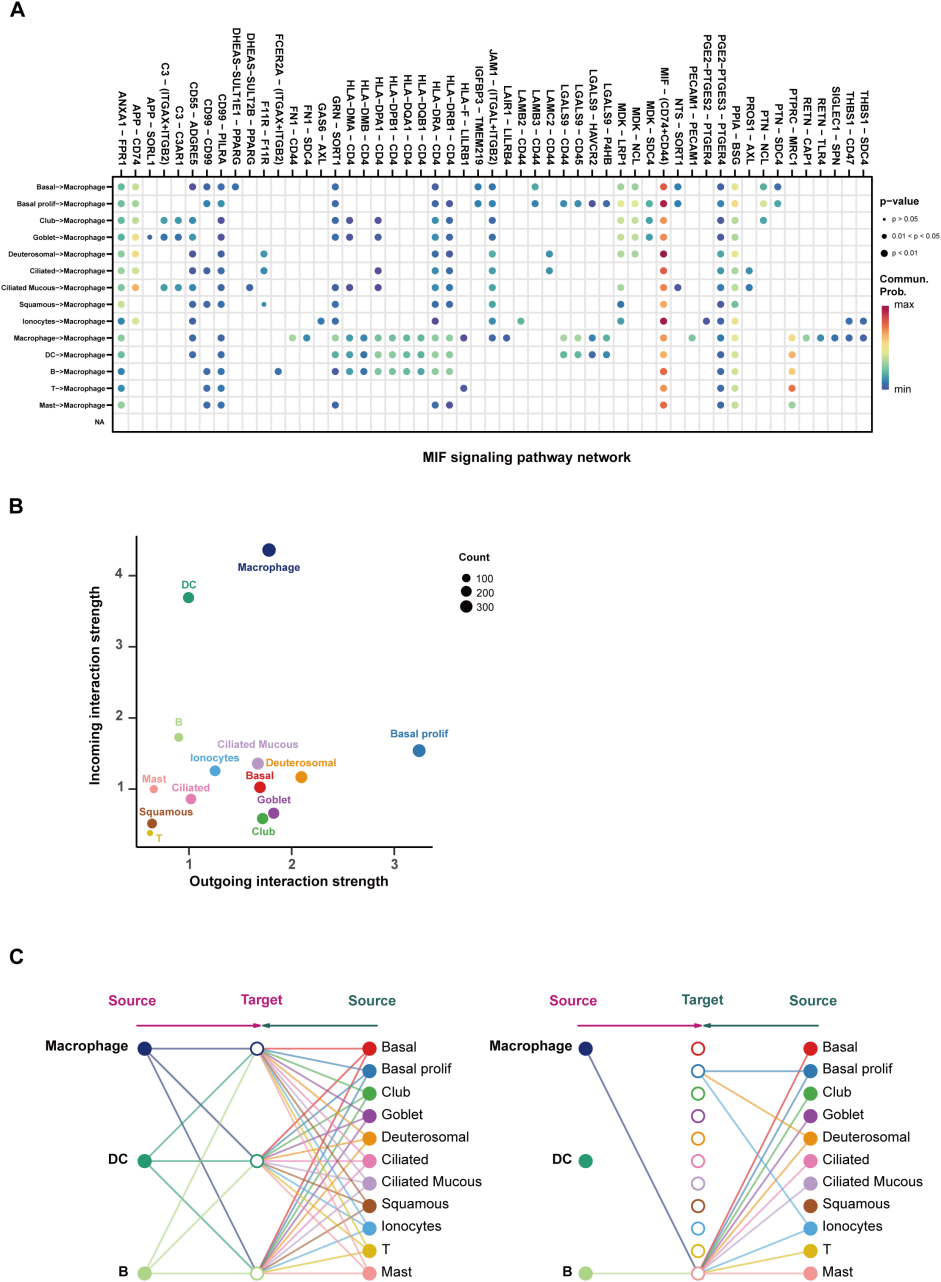
The MIF signaling axis acts as a key inflammatory amplifier between epithelium and immune cells. **(A, B)** Bubble plots depicting overall incoming and outgoing interaction strengths; **(C)** Pathway-specific communication network for the MIF signaling axis.

Furthermore, to elucidate the functional localization of HLA-DPA1- and HLA-DPB1-expressing cells within the airway epithelial microenvironment and their intercellular interaction mechanisms, we conducted cell-cell communication analysis. This approach enables the reconstruction of intercellular signaling networks at single-cell resolution, providing contextual insights into the immune responses regulated by candidate key genes. The results indicated that epithelial cells primarily acted as signal senders, whereas macrophages and dendritic cells served as signal receivers (**Figures 10A, B**), suggesting that antigen-presenting cells may occupy a central position in signal integration within the asthmatic microenvironment. Frequent interactions were observed between macrophages and basal epithelial cells (**Figure 10C**), implying that these two cell types may form key functional units in the immune regulation of the airway epithelial barrier. Bubble plots of global communication strength revealed that macrophages and dendritic cells exhibited higher incoming interaction strength compared to other cell types (**Figures 11A, B**), further supporting their central role in the immune signaling network. In the MIF signaling pathway, multiple epithelial subpopulations were predicted to transmit signals to macrophages, dendritic cells, and B cells (**Figure 11C**), suggesting that the MIF signaling axis may serve as an important bridge for communication between epithelial and immune cells. Given the limited sample size of the single-cell dataset, these findings should be interpreted as exploratory observations.

In summary, pseudotime trajectory analysis revealed the existence of a continuous functional state transition of macrophages within the childhood asthma microenvironment, with HLA-DPA1 and HLA-DPB1 showing upregulated expression during this dynamic process and being closely associated with immune-metabolic functional status. Cell-cell communication analysis further identified a signaling reception network centered on macrophages and dendritic cells, as well as the potential mechanism by which epithelial cells regulate immune cell function via the MIF signaling axis. Together, these results provide evidence at single-cell resolution that antigen-presenting cells, particularly macrophages, not only represent an important cellular context for the expression of HLA-DPA1 and HLA-DPB1 but may also serve as key functional mediators through which these genes participate in immune regulation in asthma.

## Discussion

Research has consistently linked PA to widespread dysregulation of immune regulatory networks, including but not limited to classical immune checkpoint, implicating these molecules in core pathologies like Th1/Th2 imbalance, macrophage dysfunction, and epithelial impairment (9, 12, 51). Based on multi-omic transcriptomic evidence, this study identifies HLA-DPA1 and HLA-DPB1 as candidate key genes in childhood asthma for the first time, revealed their significantly downregulated expression patterns and high diagnostic value in affected children. Functional enrichment and immune infiltration analyses further demonstrate that the downregulation of these two genes is closely associated with impaired antigen presentation, enhanced metabolic dysfunction, and infiltration of Th2-type inflammatory cells Accordingly, we propose a working hypothesis that childhood asthma may involve a state of immune-metabolic dysregulation tentatively termed “high-metabolism, low-presentation”.

Single-cell analysis further uncovered functional heterogeneity in macrophages in childhood asthma, with the Macro2 subset (metabolic/stress-related) exhibiting high expression of HLA-DPA1/DPB1. Pseudotime trajectory analysis suggested a trend of macrophage transition from an immune-activated state toward a metabolically imbalanced state. Cell–cell communication analysis delineated an aberrantly active network in which epithelial cells acted as signal senders, while macrophages and dendritic cells served as central signal receivers, with the MIF signaling axis emerging as a key bridge. It should be noted that the following single−cell results are derived from only two samples and are presented as preliminary exploratory observations. Consistent downregulation of HLA-DPA1/DPB1 expression was validated in both an *in vitro* IL-13-stimulated bronchial epithelial cell model and peripheral blood mononuclear cell samples from pediatric asthma patients. We fully acknowledge that airway epithelial cells and PBMCs represent distinct immune compartments, and direct equivalence is not appropriate. Nonetheless, using PBMCs for validation is justified: immune status changes in the airway epithelium are systemically reflected in circulating immune cells, as supported by studies demonstrating consistent expression patterns of immune-related molecules across the two compartments (52, 53). Collectively, this study establishes a novel framework of immune-metabolic dysregulation characterized by a “high-metabolism, low-presentation” state in childhood asthma, and reveals a previously unrecognized molecular and cellular mechanism centered on antigen presentation and metabolic heterogeneity of macrophages.

Our prediction results revealed that the HLA class II region, particularly HLA-DPA1 and HLA-DPB1, is significantly associated with childhood asthma, validating the GWAS findings in Asian populations (54, 55). The mechanism underlying downregulation of HLA-DPA1 and HLA-DPB1 remains unclear. Our bioinformatic analyses offer testable hypotheses: GWAS variants within HLA-DPA1 (rs111789468, rs35449774, rs3130169) are significantly associated with childhood asthma and may affect gene regulation. Transcription factors including CIITA, RFX5, and NF-Y family members, as well as miRNAs such as miR-146a and miR-155, are predicted to target these genes. Direct experimental validation (e.g., ChIP, luciferase reporter assays, and miRNA manipulation) is required to establish causality.

Through multi-algorithm machine learning screening and independent validation, this study identified HLA-DPA1 and HLA-DPB1 as candidate key genes, consistently downregulated genes in PA. These genes encode the α and β chains of MHC class II molecules, essential components for the presentation of exogenous antigens to CD4^+^ T cells by antigen-presenting cells, and can themselves be regarded as critical “checkpoints” initiating adaptive immunity (56, 57). Crucially, the dysfunction of these candidate key genes constitutes a primary event in immune dysregulation, aligning with prior evidence (16). Our findings align with earlier suggestions of potential impairment in the MHC class II pathway in severe asthma (58). Intriguingly, GSEA revealed that the downregulation of HLA-DPA1/DPB1 showed a significant negative correlation with classical pro-inflammatory pathways but a positive correlation with fundamental metabolic pathways, which is consistent with the emerging concept of immunometabolic reprogramming in chronic inflammatory diseases (58). This suggests the existence of a unique “high-metabolism, low-presentation” state within the PA airway microenvironment. On one hand, weakened antigen presentation may lead to aberrant immune recognition or tolerance breakdown. On the other hand, immune cells appear to be in a state of metabolic hyperactivity. This apparent “mismatch” between immunity and metabolism may represent a mechanism driving chronic inflammation and airway cell dysfunction. While prior studies have indicated that metabolic reprogramming profoundly influences immune cell phenotypes (59), our results directly link this concept to a specific antigen-presentation defect in PA.

We observed significantly increased infiltration of eosinophils and resting mast cells in the PA group, which aligns with the classical immunopathology of severe and allergic asthma and further supports the classic Th2-driven inflammation paradigm (60, 61). Correspondingly, the proportion of activated dendritic cells (DCs), the central antigen-presenting cells for initiating adaptive immunity, was markedly reduced (62). The diminished activation state of DCs, coupled with the downregulation of HLA-DP molecules, likely contributes to a broad impairment in antigen presentation. More importantly, correlation analysis revealed that the expression levels of HLA-DPA1 and HLA-DPB1 were both significantly negatively correlated with the abundance of eosinophils and resting mast cells. This strongly suggests that the downregulation of these candidate key genes is not merely an epiphenomenon but may be causally linked to the expansion of eosinophil/mast cell-driven inflammation. Specifically, the defect in antigen presentation could lead to the excessive activation and recruitment of these effector cells through mechanisms yet to be fully defined, such as impaired regulatory T cell function or aberrant Th2 cell differentiation. Notably, although MHC class II molecules are classically associated with type-2 immune activation in asthma, their downregulation observed in this study does not necessarily contradict enhanced type-2 inflammation. Type-2 responses reflect Th2 and eosinophil expansion, whereas MHC II denotes APC antigen-presenting capacity, which can be independently regulated. Consistent with previous studies showing that MHC II expression and APC maturation are dynamically modulated in allergic inflammation (63, 64), our data demonstrate increased eosinophils and mast cells but reduced activated DCs and HLA-DP expression in metabolically skewed macrophages. These findings suggest impaired antigen presentation despite sustained type-2 activity. Thus, decreased HLA-DPA1/DPB1 likely reflects APC functional remodeling rather than reduced immune activation. Interestingly, no significant correlation was observed between the expression of HLA-DPA1/HLA-DPB1 and the abundance of regulatory T cells (Tregs). In addition, ligand–receptor analysis did not identify CD4-related signaling interactions involving T cells. These findings suggest that the immune regulatory effects associated with these genes may not primarily involve classical CD4^+^ T-cell–mediated pathways in the analyzed dataset.

We propose a multi-layered, integrative framework for the pathogenesis of PA. The initiating event may be the downregulation of HLA-DPA1/DPB1 expression in airway mucosal immune cells, driven by genetic or environmental factors. This downregulation initiates a cascade with dual consequences. First, it directly impairs MHC class II antigen presentation, potentially affecting the precise initiation and regulation of immune responses and leading to tolerance defects (65). Second, it triggers compensatory or dysregulated cellular metabolic reprogramming, establishing an abnormal “high-metabolism, low-presentation” state.

To dissect the roles of candidate key genes at cellular resolution, we performed single-cell RNA sequencing analysis. This analysis confirmed that HLA-DPA1/DPB1 were predominantly expressed in macrophages, dendritic cells (DCs), and B cells, consistent with their antigen-presenting functions (66). Focusing on macrophages, subclustering revealed functional heterogeneity and polarization dysregulation in PA, aligning with prior studies (49, 67). Two functionally distinct subsets were identified: Macro1, skewed toward pathogen recognition and inflammation resolution, and Macro2, enriched for ion channel activity and metabolic stress pathways. In this context, the functional divergence between Macro1 and Macro2 macrophage states further underscores the immune complexity within the pediatric airway microenvironment. However, direct correspondence between these macrophage phenotypes and established clinical endotypes cannot be determined from the current dataset and warrants further investigation.

More critically, pseudotime analysis stratified macrophages into two state clusters based on HLA-DPA1/DPB1 expression: C1 (low HLA-DP, high metabolism/stress) and C2 (high HLA-DP, immune activation). In the PA microenvironment, macrophages exhibited a trend toward the C1 state. This suggests a phenotypic shift in airway macrophages, from a “sentry” phenotype (C2) adept at antigen presentation and immune activation toward a “dysfunctional” phenotype (C1) with attenuated antigen presentation but heightened metabolic stress and pro-inflammatory potential. This repolarization likely constitutes a core mechanism by which macrophages perpetuate and amplify inflammation (68).

Cell-cell communication analysis further delineated an aberrantly active signaling network. We found that airway epithelial cells (particularly basal cells) served as the dominant signal senders, while macrophages and DCs functioned as central receiving and integration hubs. This reframes the airway epithelium from a passive barrier or damage target into an active “instigator” of immune inflammation (69). Notably, the MIF signaling pathway was widely activated between epithelial cells and macrophages/DCs/B cells. As a pleiotropic pro-inflammatory cytokine, MIF can inhibit macrophage migration from inflammatory sites and enhance their release of inflammatory mediators, thereby creating a positive feedback loop that sustains inflammation (70). The hyperactivation of this pathway may represent a key molecular bridge connecting epithelial cell activation to the persistent inflammatory state of myeloid cells, forming a self-reinforcing inflammatory circuit.

This altered state, in turn, drives the polarization of macrophages toward the C1 (metabolic/stress) phenotype, rendering them dysfunctional. Simultaneously, injured or stimulated airway epithelial cells become activated, transitioning from a passive barrier to an active broadcaster of alarm and danger signals into the microenvironment. Macrophages and dendritic cells, serving as signaling hubs, are themselves compromised by pre-existing HLA-DP downregulation and metabolic disturbance. Upon receiving these potent epithelial-derived signals, their inflammatory state becomes locked and amplified. Upon receiving strong epithelial signals, the outgoing communication from macrophage/DC hubs was markedly enhanced, establishing a self-perpetuating inflammatory circuit that is epithelium-initiated and centered on myeloid activity (71).

This aberrant epithelial-myeloid communication, particularly through positive feedback loops like the MIF pathway, ultimately fuels the recruitment and activation of effector cells (e.g., eosinophils, mast cells) and leads to chronic airway inflammation and remodeling (72). Our immune infiltration analysis, showing a negative correlation between HLA-DP expression and eosinophil/mast cell infiltration, provides corroborative evidence for this framework.

Furthermore, our findings hold notable translational implications. HLA-DPA1 and HLA-DPB1 may serve not only as potential diagnostic biomarkers but also as nodes within a therapeutically targetable “antigen presentation-metabolism” axis. For instance, strategies aimed at restoring normal antigen-presenting function in macrophages or correcting their metabolic dysregulation, as well as agents targeting key nodes in the epithelium-macrophage dialogue (such as the MIF-CD74 axis), could provide novel intervention avenues for PA subtypes that respond poorly to existing therapies (73).

This study has several limitations, but overall provides a testable multi-level transcriptomic framework. First, the bulk transcriptome dataset used for machine learning screening has a large sample size, whereas the single-cell RNA-seq dataset (GSE254127) contains only two airway samples, which cannot fully capture the individual heterogeneity of the pediatric airway epithelial cell composition. Second, although IL-13 stimulation experiments in BEAS-2B cells and validation in patient PBMC samples provide complementary evidence at the local airway and systemic levels, the molecular mechanisms and local expression patterns underlying the downregulation of HLA-DPA1 and HLA-DPB1 still require direct experimental verification in the future. Importantly, the “high metabolism, low presentation” hypothesis we propose is consistent with the transcriptomic data but has not been tested by functional perturbation experiments; therefore, we define HLA-DPA1/DPB1 as “candidate key genes” rather than definitive regulators. Future studies employing gain- or loss-of-function approaches in macrophages or airway epithelial cells are needed to determine whether the downregulation of HLA-DPA1/DPB1 acts as a driver or a consequence of immunometabolic dysregulation in childhood asthma.

## Conclusion

In this study, we employed an integrative strategy combining multi-level transcriptomic analysis and machine learning to identify HLA-DPA1 and HLA-DPB1 as candidate key genes in childhood asthma. Functional characterization associated their downregulation with impaired antigen presentation and enhanced metabolic dysfunction, leading us to propose a “high-metabolism, low-presentation” immune-metabolic dysregulation framework. Single-cell analysis further uncovered functional heterogeneity among macrophages, with a distinct subset (Macro2) exhibiting high HLA-DPA1/DPB1 expression and a pseudotime trajectory indicating a shift from immune activation toward metabolic imbalance. Cell-cell communication analysis highlighted an aberrantly active epithelial-myeloid signaling network centered on the MIF axis, with epithelial cells serving as predominant signal senders and macrophages/dendritic cells as central receivers. The downregulation of HLA-DPA1 and HLA-DPB1 was validated in both IL-13-stimulated bronchial epithelial cells and peripheral blood mononuclear cells Collectively, these findings position HLA-DPA1 and HLA-DPB1 as key players linking antigen presentation defects, macrophage polarization, and aberrant epithelial-immune crosstalk in childhood asthma, providing new mechanistic insights and identifying potential diagnostic biomarkers and therapeutic targets that warrant further validation in larger cohorts and functional investigations to support clinical translation.

## Data Availability

The datasets presented in this study can be found in online repositories. The names of the repository/repositories and accession number(s) can be found in the article/**Supplementary Material**.

## Supplementary material

Supplementary Figure 1 Identification of immune-related candidates in the validation cohort GSE65204. **(A)** Volcano plot of the top 10 DEGs ranked by |log2FC|; **(B)** Heatmap of the top 10 DEGs ranked by |log2FC|; **(C)** Venn diagram of the intersection between DEGs and ICRGs.

Supplementary Figure 2 Machine learning screening in GSE65204. **(A)** Gene selection by LASSO regression model; **(B)** Gene selection by SVM-RFE; **(C)** Feature importance ranking by XGBoost; **(D)** UpSet plot of consensus genes selected by LASSO, SVM−RFE, and XGBoost.

Supplementary Figure 3 Genomic characterization and functional profiling of candidate key genes. **(A)** Chromosomal localization of HLA-DPA1 and HLA-DPB1; **(B)** Subcellular localization of HLA-DPA1 and HLA-DPB1; **(C)** Functional coherence of HLA-DPA1 and HLA-DPB1; **(D)** Co-expression analysis of HLA-DPA1 and HLA-DPB1.

Supplementary Figure 4 Dissecting the multilayer transcriptional and post-transcriptional regulation of candidate key genes. **(A)** Predicted miRNA targeting of HLA-DPA1 and HLA-DPB1; **(B)** miRNA-mRNA regulatory network; **(C)** TF-mRNA regulatory network; **(D)** TF-mRNA-miRNA regulatory network.

Supplementary Figure 5 Quality control of single-cell atlas in the pediatric asthma. **(A)** ScRNA-seq data before and after quality control; **(B)** Variance plot illustrating gene expression variation across all PA cells, with red dots denoting highly variable genes and black dots representing non-variable genes; **(C)** PCA identifying the top 30 PCs.

Supplementary Figure 6 Expression of HLA-DPA1 and HLA-DPB1 along the pseudotime trajectory.

Supplementary Table 1 282 immune checkpoint-related genes (ICRGs) used in this study.

Supplementary Table 2 List of DEGs in PA.

Supplementary Table 3 Candidate genes identified from the intersection of DEGs and ICRGs.

Supplementary Table 4 GO and KEGG enrichment analysis of the 39 candidate genes.

Supplementary Table 5 Full list of genes identified by each machine learning algorithm.

Supplementary Table 6 Candidate genes identified from the intersection of DEGs and ICRGs in GSE65204.

Supplementary Table 7 GSEA enriched pathways of candidate key genes.

Supplementary Table 8 List of oligos used in this study.

Supplementary Table 9 Clinical features and prognosis of the six pediatric asthma patients and three healthy donors.
