# Supplementary figures and images for: Integrative multi-omics reveals that downregulation of HLA-DPA1/DPB1 drives macrophage immune-metabolic dysregulation in pediatric asthma
Source: Front Immunol. 2026 Jun 3;17:1835475. doi: 10.3389/fimmu.2026.1835475 (PMC13272086; doi:10.3389/fimmu.2026.1835475)

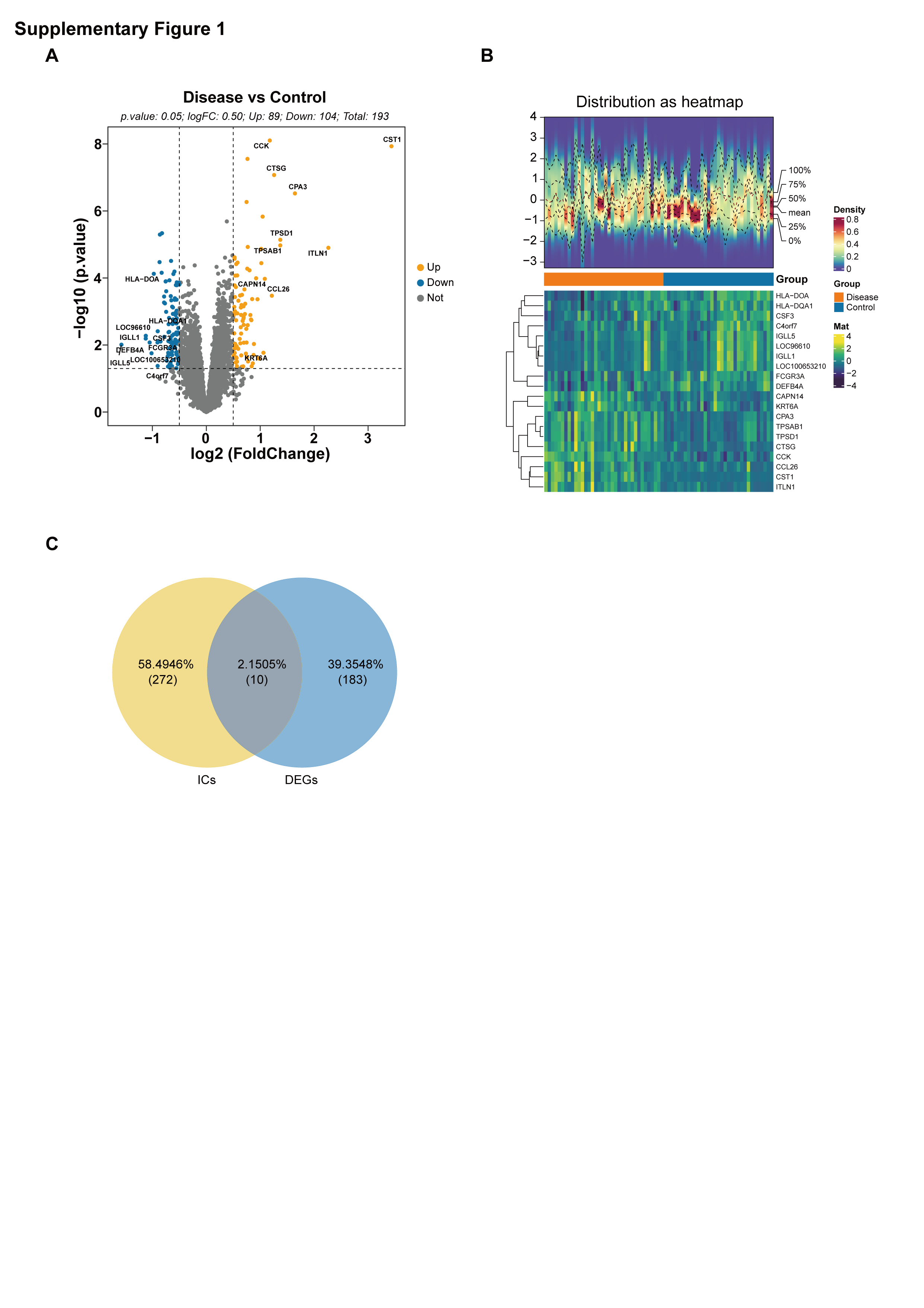

Supplement: Supplementary Figure 1 — Identification of immune-related candidates in the validation cohort GSE65204. (A) Volcano plot of the top 10 DEGs ranked by |log2FC|; (B) Heatmap of the top 10 DEGs ranked by |log2FC|; (C) Venn diagram of the intersection between DEGs and ICRGs. [file Image1.tif]

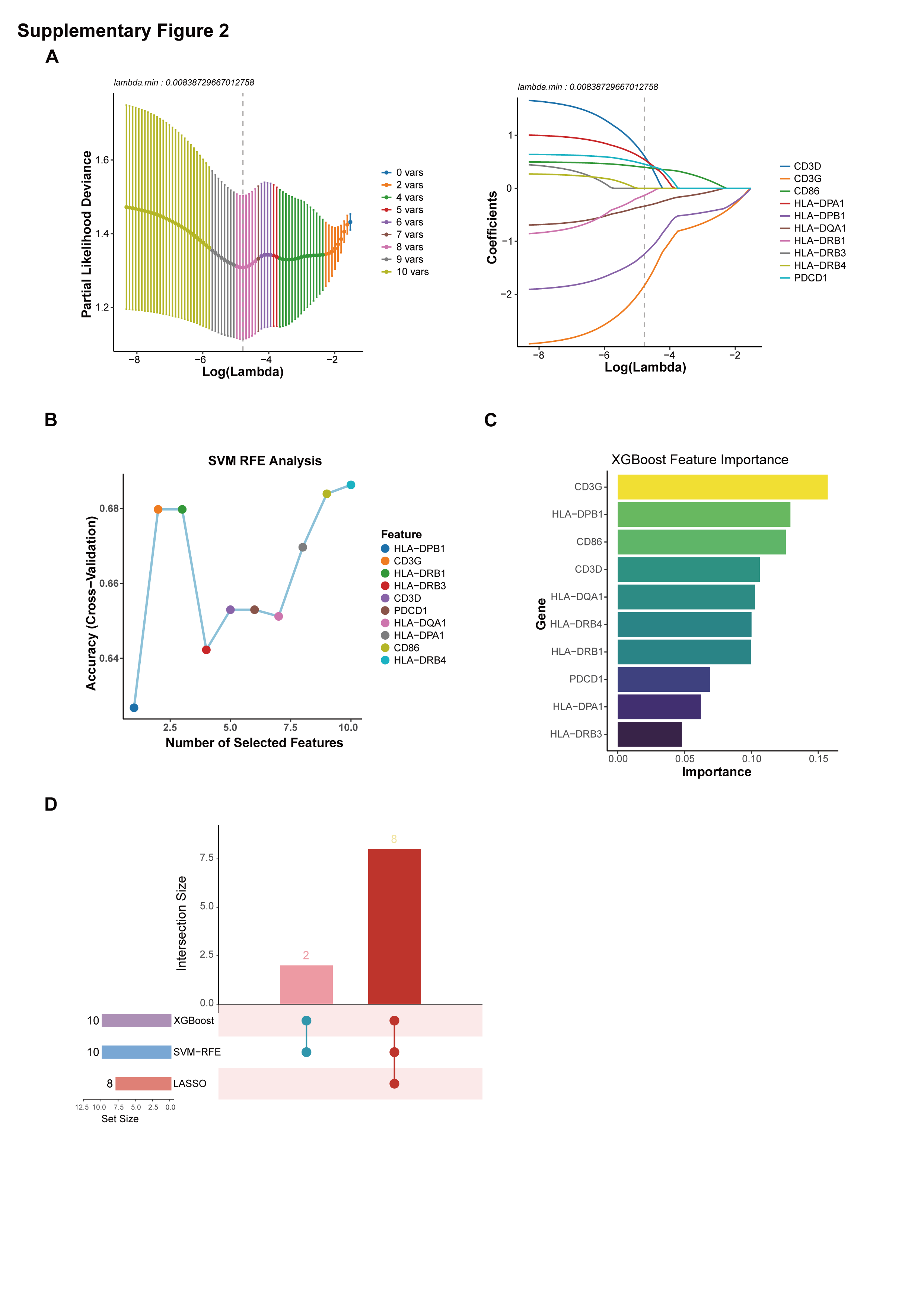

Supplement: Supplementary Figure 2 — Machine learning screening in GSE65204. (A) Gene selection by LASSO regression model; (B) Gene selection by SVM-RFE; (C) Feature importance ranking by XGBoost; (D) UpSet plot of consensus genes selected by LASSO, SVM−RFE, and XGBoost. [file Image2.tif]

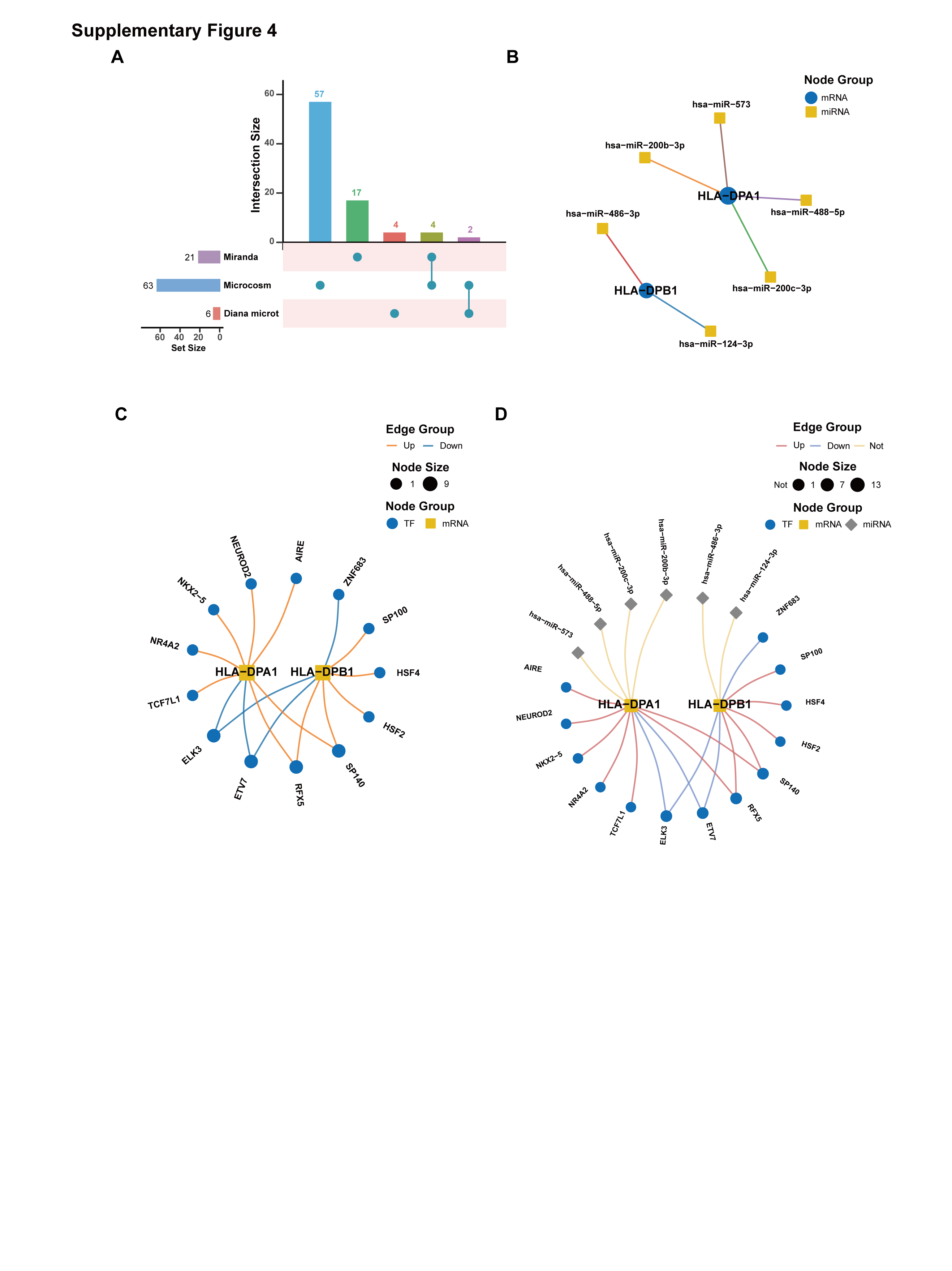

Supplement: Supplementary Figure 4 — Dissecting the multilayer transcriptional and post-transcriptional regulation of candidate key genes. (A) Predicted miRNA targeting of HLA-DPA1 and HLA-DPB1; (B) miRNA-mRNA regulatory network; (C) TF-mRNA regulatory network; (D) TF-mRNA-miRNA regulatory network. [file Image4.tif]

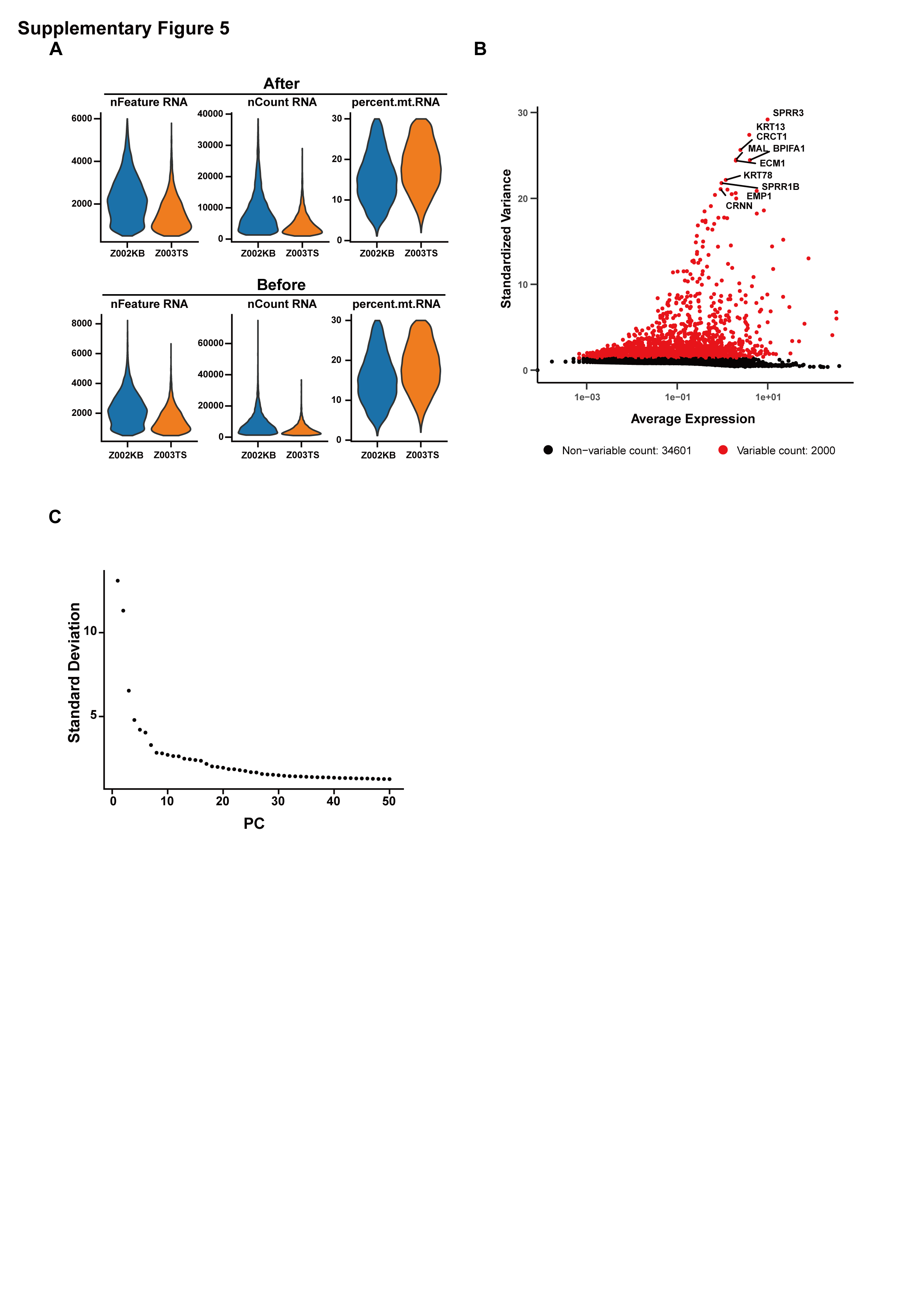

Supplement: Supplementary Figure 5 — Quality control of single-cell atlas in the pediatric asthma. (A) ScRNA-seq data before and after quality control; (B) Variance plot illustrating gene expression variation across all PA cells, with red dots denoting highly variable genes and black dots representing non-variable genes; (C) PCA identifying the top 30 PCs. [file Image5.tif]

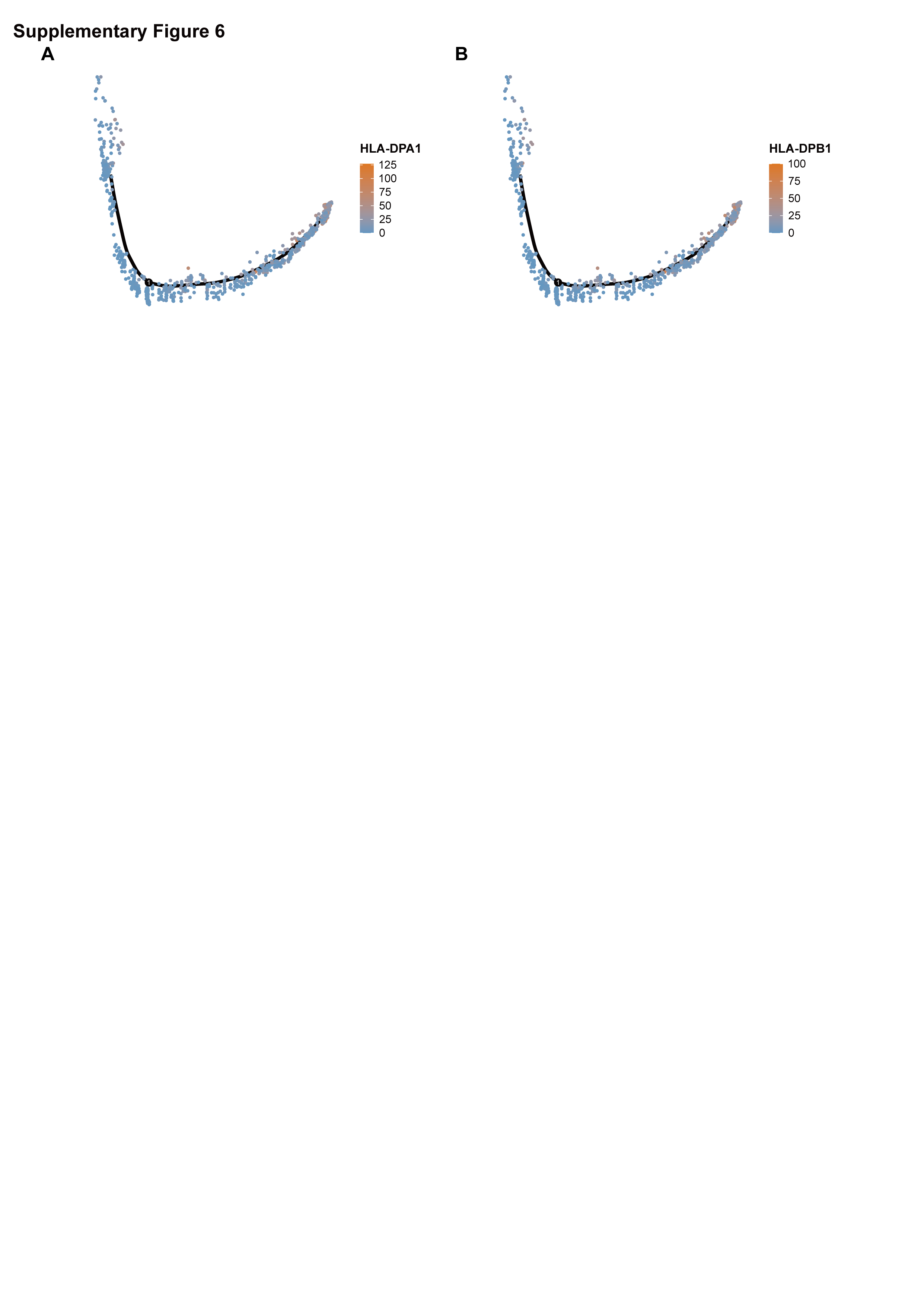

Supplement: Supplementary Figure 6 — Expression of HLA-DPA1 and HLA-DPB1 along the pseudotime trajectory. [file Image6.tif]

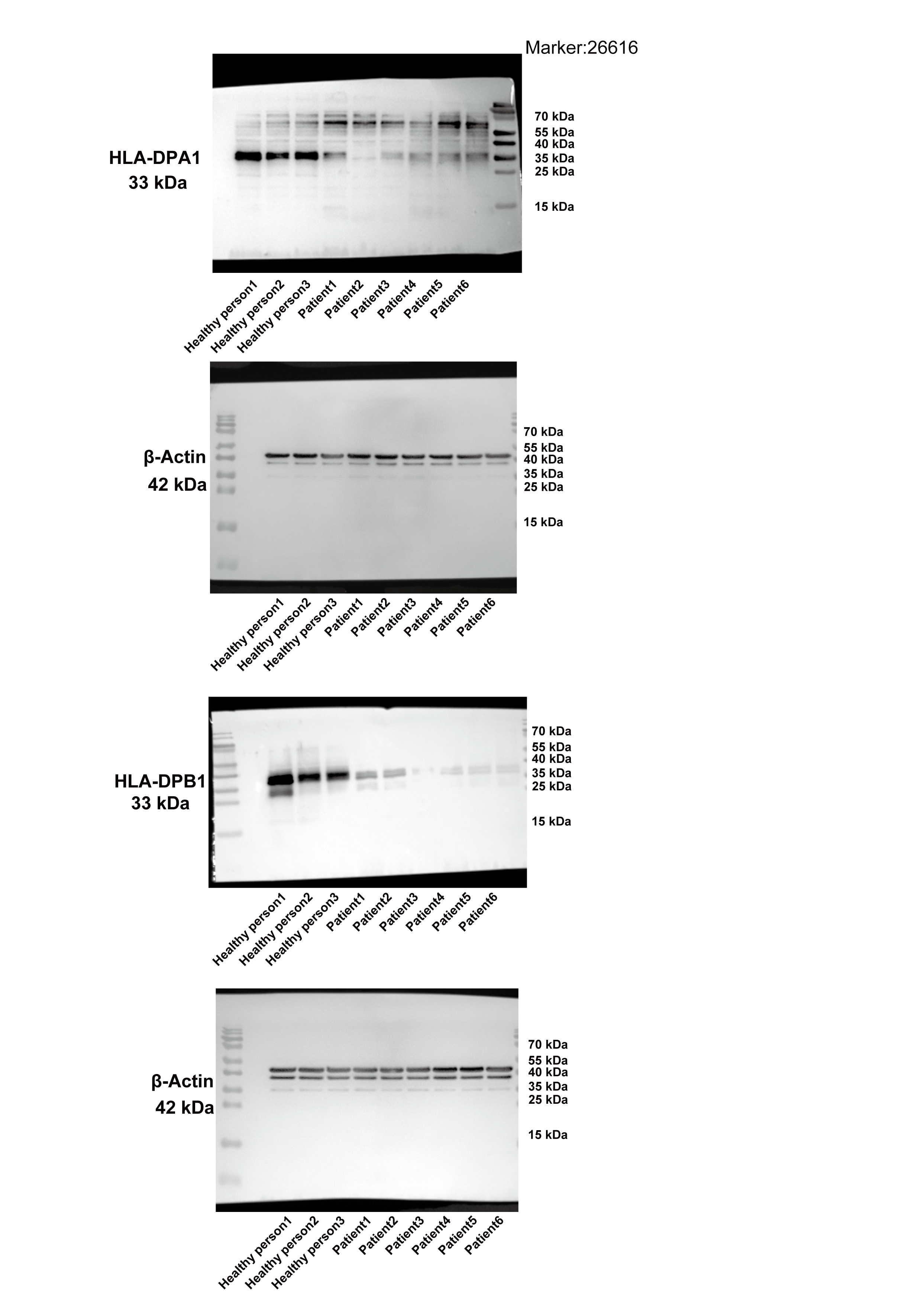

Supplement: Supplementary file 6 [file Image7.tiff]

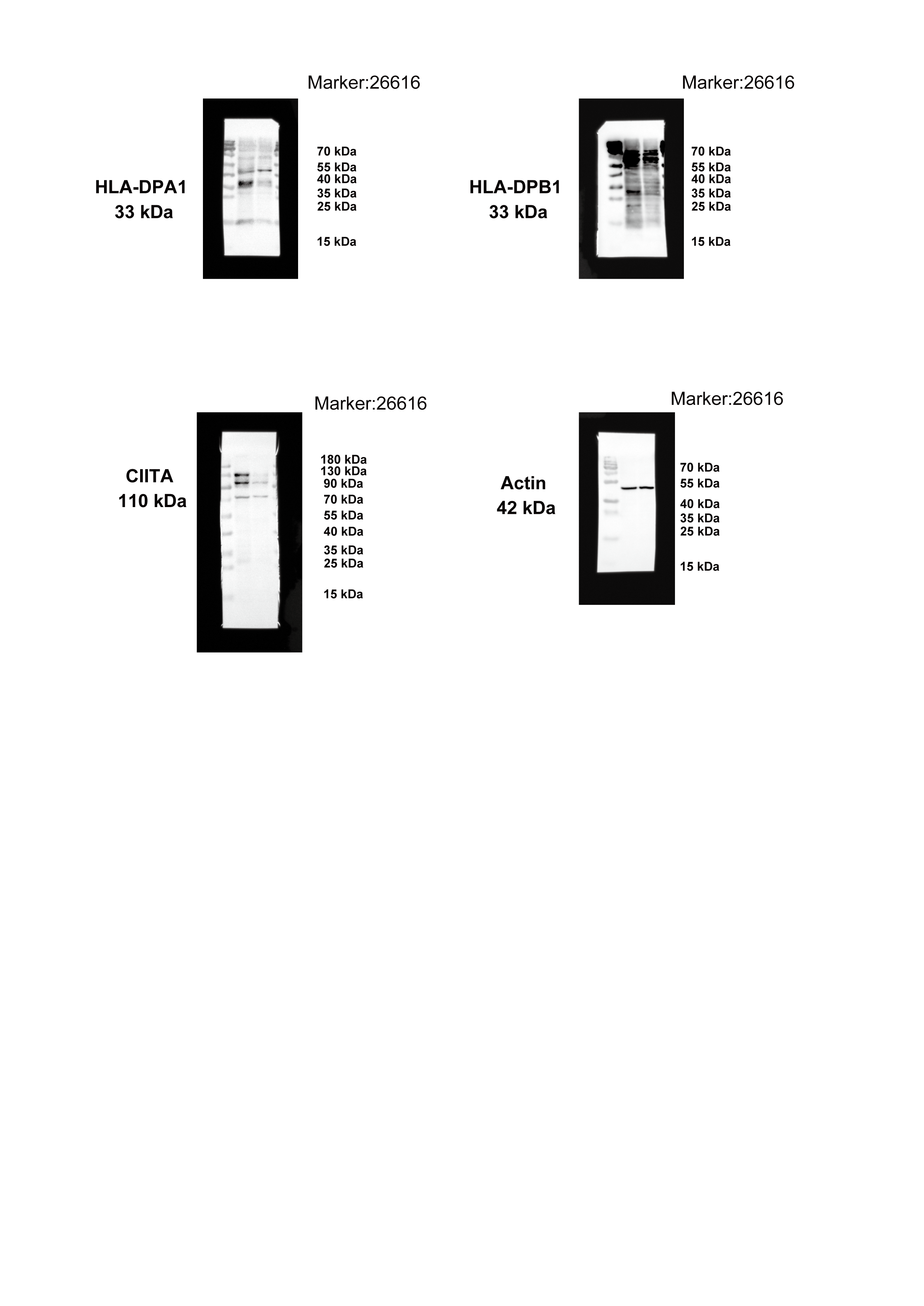

Supplement: Supplementary file 7 [file Image8.tif]
