# Supplementary material for: Integrative multi-omics reveals that downregulation of HLA-DPA1/DPB1 drives macrophage immune-metabolic dysregulation in pediatric asthma
Source: Front Immunol. 2026 Jun 3;17:1835475. doi: 10.3389/fimmu.2026.1835475 (PMC13272086; doi:10.3389/fimmu.2026.1835475)
Supplement: Supplementary file 20 [file Table2.docx]

**Supplementary table 8: List of oligos used in this study**

| **Target gene** | **Oligo sequences** |
| --- | --- |
| *HLA-DPA1* | forward, 5ʹ-GGAAGCGCTGTGTCCCATTA-3ʹ  reverse, 5ʹ- AAACTGTGGGCCTCTAGCAC -3ʹ |
| *HLA-DPB1* | forward, 5ʹ-CGCGTTTAATGGGACACAGC-3ʹ  reverse 5ʹ- CTGTTCCAGTACTCCGCAGC -3ʹ |
| *GAPDH* | forward, 5ʹ-GTCATGAGTCCTTCCACGATACC-3ʹ  reverse, 5ʹ-GGAGTCCACTGGCGTCTTCA-3ʹ |
