# Supplementary material for: Integrative multi-omics reveals that downregulation of HLA-DPA1/DPB1 drives macrophage immune-metabolic dysregulation in pediatric asthma
Source: Front Immunol. 2026 Jun 3;17:1835475. doi: 10.3389/fimmu.2026.1835475 (PMC13272086; doi:10.3389/fimmu.2026.1835475)
Supplement: Supplementary file 21 [file Table3.docx]

| **Patient/HD** | **Gender** | **Age at diagnosis** | **WBC** |
| --- | --- | --- | --- |
| **No.** |  | **(years)** | **(×10^9^/L)** |
| HD1 | Male | 8 | 10.74 |
| HD2 | Female | 7 | 9.66 |
| HD3 | Male | 10 | 8.50 |
| P#1 | Female | 11 | 8.24 |
| P#2 | Male | 11 | 7.06 |
| P#3 | Male | 8 | 12.27 |
| P#4 | Male | 9 | 9.73 |
| P#5 | Female | 12 | 13.50 |
| P#6 | Female | 7 | 15.64 |

**Supplementary Table 9. Clinical features and prognosis of the six pediatric asthma patients and three healthy donors**
